# Comparative mapping in the Poaceae family reveals translocations in the complex polyploid genome of sugarcane

**DOI:** 10.1186/s12870-014-0190-x

**Published:** 2014-07-26

**Authors:** Karen S Aitken, Meredith D McNeil, Paul J Berkman, Scott Hermann, Andrzej Kilian, Peter C Bundock, Jingchuan Li

**Affiliations:** 1 CSIRO Plant Industry, Queensland Bioscience Precinct, 306 Carmody Rd, St Lucia, Brisbane 4067, QLD, Australia; 2 BSES Limited, 50 Meiers Road, Indooroopilly, Brisbane 4068, QLD, Australia; 3 Diversity Arrays P/L, 1 Wilf Crane Crescent, Yarralumla, Canberra 2600, ACT, Australia; 4 Southern Cross University, Ctr Plant Conservation Genetics, Lismore 2480, NSW, Australia

**Keywords:** Saccharum, Poaceae, Synteny, Orthology, Translocations, SNP markers

## Abstract

**Background:**

The understanding of sugarcane genetics has lagged behind that of other members of the Poaceae family such as wheat, rice, barley and sorghum mainly due to the complexity, size and polyploidization of the genome. We have used the genetic map of a sugarcane cultivar to generate a consensus genetic map to increase genome coverage for comparison to the sorghum genome. We have utilized the recently developed sugarcane DArT array to increase the marker density within the genetic map. The sequence of these DArT markers plus SNP and EST-SSR markers was then used to form a bridge to the sorghum genomic sequence by BLAST alignment to start to unravel the complex genomic architecture of sugarcane.

**Results:**

Comparative mapping revealed that certain sugarcane chromosomes show greater levels of synteny to sorghum than others. On a macrosyntenic level a good collinearity was observed between sugarcane and sorghum for 4 of the 8 homology groups (HGs). These 4 HGs were syntenic to four sorghum chromosomes with from 98% to 100% of these chromosomes covered by these linked markers. Four major chromosome rearrangements were identified between the other four sugarcane HGs and sorghum, two of which were condensations of chromosomes reducing the basic chromosome number of sugarcane from x = 10 to x = 8. This macro level of synteny was transferred to other members within the Poaceae family such as maize to uncover the important evolutionary relationships that exist between sugarcane and these species.

**Conclusions:**

Comparative mapping of sugarcane to the sorghum genome has revealed new information on the genome structure of sugarcane which will help guide identification of important genes for use in sugarcane breeding. Furthermore of the four major chromosome rearrangements identified in this study, three were common to maize providing some evidence that chromosome reduction from a common paleo-ancestor of both maize and sugarcane was driven by the same translocation events seen in both species.

## Background

Sugarcane is a member of the Poaceae family that contributes 75% of the worlds sugar supply and is being heralded as an important source as an alternative biofuel [[1]]. However, knowledge of the genetic architecture of this crop currently lags behind that of other crops of the Poaceae family such as wheat, barley and rice. It is important to uncover the genome structure of a particular crop to guide informed decisions in the identification of important genes/traits that can be utilized during breeding and germplasm introgression.

Modern sugarcane cultivars (2n = 100-120) have complex polyploid, aneuploid genomes that make classical genetic, molecular genetic, and breeding studies difficult to interpret, as information on the structure and organisation of the genome is incomplete [[2]]. They are essentially hybrids between the polyploid domesticated species, Saccharum officinarum L (2n = 80) and the polyploid wild species, *S. spontaneum* L (2n = 40-128). The cultivated hybrids have undergone a series of backcrosses to *S. officinarum* as the female parent to recover agronomically adapted genotypes with a high sugar yield. Using in situ hybridisation, [[3]] determined that the basic chromosome number of *S. officinarum* is x = 10 and for *S. spontaneum* is x = 8, with 10-15% of the genome of modern sugarcane cultivars contributed by *S. spontaneum*, 80% *S. officinarum* and 11-15% recombinant chromosomes between the two species [[4]]. Studies have shown that chromosome pairing is mostly as bivalents, although an assortment of meiotic irregularities can occur and contribute to the formation of aneuploid gametes [[5],[6]]. Sugarcane clones are propagated vegetatively and are stable over many years some cultivars have been grown commercially for over 20 years [[7]].

Genetic studies of sugarcane have been difficult due to the polyploid and highly heterozygous nature of the genome. For example, molecular markers such as restriction fragment length polymorphism (RFLPs) and simple sequence repeats (SSRs) can reveal several markers or alleles at one locus. They can be single dose (ie single copy at a particular locus), double dose (two copies) or multi dose where it is often not possible to know how many copies are at a particular locus. To date, a number of genetic maps have been constructed using single dose (SD) markers. Single dose markers are used in coupling to construct linkage groups, and multi allelic markers and markers in repulsion are used to sort these linkage groups into homology groups. The genetic maps generated to date have utilized the following markers; random amplified polymorphic DNA (RAPDs) [[8],[9]], RFLPs [[10]-[13]] and amplified fragment length polymorphism (AFLPs) [[14]-[16]] and simple sequence repeats (SSRs) [[17],[18]]. Even the most extensive of these genetic maps still only covers approximately half of the sugarcane genome [[15]]. Recently, the development of a high-throughput marker system, diversity arrays technology (DArTs), has enabled the creation of medium-density genetic maps for plants with complex genomes and limited sequence information available [[19]]. DArT arrays generate whole genome fingerprints by scoring the presence versus absence of DNA fragments in genomic representations generated from genomic DNA samples through the process of complexity reduction [[20]]. DArT maps have now been generated for many plants in the grass family, including rice [[20]], barley [[19]], wheat [[21]] and sorghum [[22]]. In an effort to increase the marker density for sugarcane, a diversity array was developed using a combination of *S. officinarum* and *S. spontaneum* accessions and sugarcane cultivars [[23]]. This has resulted in a medium density genetic map for sugarcane with enough sequence information to allow comparative mapping [[24]].

Comparative genomics is a powerful tool that can provide a starting point for accelerating progress in revealing the genomic structure of crops that are lacking in the necessary genomic tools [[25]]. Aligning markers or partial sequences to fully sequenced genomes enables researchers to deduce information about gene content and order of a particular crop of interest. Sugarcane is a member of the Andropogoneae tribe, within the grass family Poaceae, along with other tropical grasses such as sorghum (*Sorghum bicolor* L. Moench), maize (*Zea mays*) and rice (*Oryza sativa*). Evolutionary studies have speculated that sorghum and maize may have diverged from a common ancestor about 29 million years ago [[26]] after which maize underwent polyploidation and subsequent reorganisation of the genome. In contrast, rice and the maize/sorghum lineage may have diverged from a common ancestor around 46 million years ago and show much greater levels of chromosome structural rearrangement [[26]]. Whilst, sugarcane and sorghum may have shared a common ancestor less than 10 million years ago and retain very similar gene order [[27],[28]]. This close evolutionary relationship has led to the use of sorghum as a model crop for comparative genetic studies to gain a greater understanding of the genome of sugarcane [[25]]. The availability of the complete genome sequence of rice, maize and more recently sorghum, and the close evolutionary history of these species, has allowed some powerful comparative genomic studies [[25],[26],[29]] to be undertaken to gain an understanding of the genetic architecture of other crops where genomic sequence is lacking.

In this current study we have taken advantage of the development of a new, high-throughput marker system (DArTs) to increase the marker density combined with the addition of SNP and EST-SSR markers. This has led to an increase in genome coverage which will aid in the identification of QTL regions containing agronomically important genes of interest for sugarcane breeders. Furthermore, the availability of the genome sequence of maize, rice and more specifically sorghum has allowed a detailed study of the syntenic relationships between these crops. The generation of this denser genetic map will ultimately lead to a greater understanding of the genetic architecture of the sugarcane genome which had previously been lacking. This understanding will now allow the interrogation of important regions and identification of target genes for breeding.

## Results

### Assembly of Saccharum linkage groups into homo(eo)logous groups (HGs)

The genetic linkage map of Q165 presented in Additional file 1 was arranged into eight HGs which represents the lowest *Saccahrum* basic chromosome number (x = 8). Initially this was carried out using the multiple allelic loci detected by single SSR, RFLP or SNP markers. The location of the LGs was then verified using sequence information from all the 677 markers with a known sequence that had a primary correspondence to the sorghum genome at a significance level of P < e^−20^. This allowed the 160 LGs to be condensed into the eight HGs (Table 1). Cultivar Q165 has a total of 110 chromosomes of which 15–17 are inherited from *S. spontaneum*, 82–83 are inherited from *S. officianrum* and 11–12 are recombinant between the two species [[30]].

**Table 1:** **Number of single dose DArT markers mapped in each HG and length of HG in the Q165 linkage map and aligned to the sorghum genome (see Additional file**1**for details)**

**HG**	**Sorghum chromosome**	**No. LG**	**No. DART**	**Total number of markers mapped**	**Length of HG in cM**
4	Sb1	19	125	308	1217.2
8	Sb8 Sb2	24	134	297	1319.2
3	Sb3	20	75	218	1243.6
1	Sb4	19	125	282	1253.4
2	Sb5 Sb6	23	198	475	1818.0
5	Sb7	13	94	222	846.6
6	Sb9	18	111	232	678.3
7	Sb10	17	90	204	1020.5
unassigned		18	21	45	395.9
total		168	976	2283	9792.7

### Alignment to sorghum

**Figure 1:**
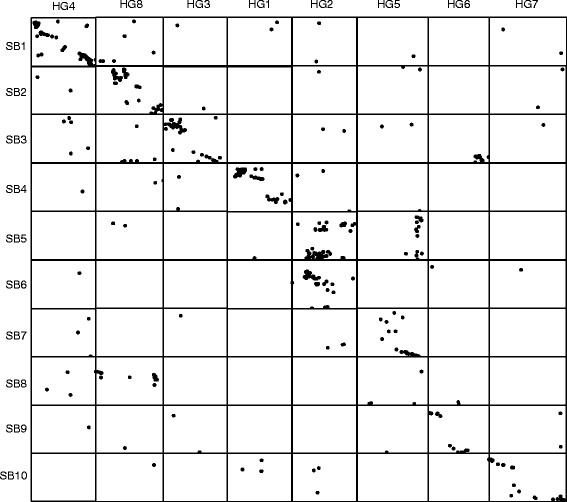
**Global distribution of synteny between the 10 sorghum chromosome sequence (SB1-10) and the 8 composite linkage groups from*Saccharum*cultivar Q165.** Loci showing homology between the two genomes at P < 1e^−20^ significance threshold are indicated by dots.

The sequence information from the DArT, SNP, RFLP and the EST derived SSR markers were used to align the sugarcane LGs to the sorghum genome sequence (Additional file 1, Table 2). This was achieved by comparing the DNA sequence of the 959 markers with a known sequence to the genome sequence of sorghum using BLASTN homology searching. At the significance threshold of P < 1 × 10^−20^ there were 677 primary correspondences and a further 175 secondary and tertiary correspondences where the sequence aligned to a further one or two sites at P < 1 × 10^−20^ but a lower significance level than the primary correspondence (Table 2). In combination with the sequence information from 677 markers, 282 alleles from SSR, SNP and RFLP loci gave a total of 959 links between the sugarcane genetic map and the sorghum genome. Using these 959 informative markers the majority of the sugarcane linkage groups could be assigned to a single sorghum chromosome with high confidence (P < 1 × 10^−20^) at the macrosyntenic level. As *S. spontaneum* has a basic chromosome number of x = 8, the 8 HGs of *Saccharum* are aligned to the ten chromosome sequences of sorghum. A composite map was produced for each HG to increase the number of markers that could be used for alignment between the *Saccharum* HGs and the sorghum genome. The sugarcane LGs within a HG where possible were condensed into single consensus LGs. This was only possible where the LGs shared two or more markers in common. The consensus LG for each HG was compared with the sorghum genome (Figure 1). In some cases the LGs could not be included as there were not enough markers in common between the LGs within the HG. For example HG6 aligned to Sb9 and 3 using the consensus sequence but when all the LGs are considered parts of LGs also aligned to Sb8 and Sb5 (Table 3). In total over 92% of the sorghum genome was covered by these composite LGs.

**Table 2:** **Number of sugarcane loci with BLASTN (P < 1 × 10**^**−20**^**) correspondences in ten sorghum chromosomes (Sb1-10)**

***Sorghum bicolor*** **chromosomes**	**Primary correspondences**	**All correspondences**	**No. of incongruous loci**	**No. with no hit to sorghum**
Sb 1	109	150	16	15
Sb 2	69	80	16	4
Sb 3	79	101	13	2
Sb 4	98	138	32	10
Sb 5	78	96	6	13
Sb 6	68	85	8	12
Sb 7	44	53	41	13
Sb 8	25	29	5	2
Sb 9	55	62	37	10
Sb 10	52	58	21	6
Total	677	852	195	87

**Table 3:** Distance covered in base pairs of the sorghum genome sequence by the mapped markers

**Sugarcane HG**	**Sorghum chromosome**	**Distance covered (bp)**	**Percent of sorghum chromosome covered**	**Translocation chromosome(s)**	**Distance covered (bp)**	**Percent of chromosome covered by markers**
HG1	**4**	61181-68024049	99.0%	10	49989490-68023941	29.6%
HG2	**5**	1921299-62103178	96.5%	**6**	1820265-61619875	96.1%
HG3	**3**	42138-74366564	99.8%			
HG4	**1**	175578-73559859	99.4%			
HG5	**7**	724155-63641050	97.8%	5	380277-61719143	95.3%
HG6	**9**	407317-58839141	98.0%	3	463958-38224709	50.1%
				8	633124-7753209	12.8%
				5	31121104-62153593	49.8%
HG7	**10**	1611962-60601826	96.7%			
HG8	**2**	652374-77373466	98.4%	**8**	33432881-55441494	40%

### Interchromosomal rearrangements between Saccharum and sorghum chromosomes

#### HG1

When all the LGs within this HG are combined 99% of Sb4 is covered by this HG (Table 3). This HG is highly syntenic to Sb4 with 80% of the markers with sequence hits of e-values ≤ ^−20^ aligning to Sb4. Only on LG8 did there appear to be an interchromosomal rearrangement with 11 markers that had homology to Sb10 inserted at the end of the LG (Additional file 1; Table 3), this same LG has an intrachromosomal rearrangement.

#### HG2

HG2 aligned to Sb5 and Sb6 with 8 LGs containing markers aligned to both chromosomes. Of all the markers with a known sequence, over 90% hit these chromosomes indicating a high level of synteny. When a composite LG was created from the LGs over 96% of Sb5 and Sb6 were covered by this HG (Table 3). The LGs that demonstrated synteny with both chromosomes could be explained in the most part by one translocation event although LG1a exhibited a more complex rearrangement that was supported by gene sequence markers (Additional file 1).

#### HG3

HG3 was highly syntenic to Sb3 and the consensus LG covered 99.8% of this sorghum chromosome (Table 3, Additional file 1). Of the markers with known sequence only LG 10 contained markers that had significant homology to another sorghum chromosome (Sb4).

#### HG4

HG4 was syntenic to Sb1 and the consensus LG covered 99.4% of this chromosome (Table 3, Additional file 1). Only LG1b contained any significant markers that had homology to other chromosomes and as they were at the end of the LG could be due to spurious linkage.

#### HG5

HG5 aligned to two sorghum chromosomes Sb7 and Sb5. The coverage was over 90% for both chromosomes. Five of the 13 LGs contained significant homology to both chromosomes and LGs 46, 47, 52 and 84 all represented a single translocation event at a similar position in the LG (Additional file 1, Table 3). LG52 also contain the lower part of Sb8 but more markers are needed to verify this.

#### HG6

HG6 aligned to Sb9 but contained a large number of markers that aligned to different chromosomes. Of the markers that had known sequence 67% mapped to other chromosomes than Sb9, including Sb3, Sb8 and Sb5. When the composite LG was considered, 98% of Sb9 was covered, 50% of Sb3 and Sb5 and 12% of Sb8 (lower part) (Table 3, Additional file 1). Although the majority of the LGs seemed to contain a backbone of Sb9, LG 20, 17 and 1 all contained a region that had homology to Sb8. In addition LG20 contained a group of markers with homology to Sb3 which appeared from a single translocation event at the end of Sb9.

#### HG7

This HG aligned to Sb10 with the composite LG covering 97% of the chromosome (Table 3, Additional file 1). Although a number of the markers did align to different chromosomes (38%) there was no real pattern observed. This could be due to low marker coverage as this HG contained the smallest number of markers of any of the HGs (Additional file 1).

#### HG8

This HG aligned to Sb8 and Sb2 and the composite LGs covered approximately 98% of Sb2 and 40% of Sb8 (Table 3, Additional file 1). Only LG3, 99 and 26 had markers with homology to both chromosomes. The majority of the LG (15/24) aligned only to Sb2 which is the largest sorghum chromosome. Sb8 is the smallest sorghum chromosome and only 6 LGs aligned to this chromosome. LG 99 and 26 both had translocations at the end of Sb2 but LG3 contained at least two rearrangements. Less than half of Sb8 was present in this HG, which could be due to low polymorphism levels of this chromosome or it is possible that the rest of the chromosome is present in other HGs.

### Rearrangements between Saccharum and Sorghum chromosomes

**Figure 2:**
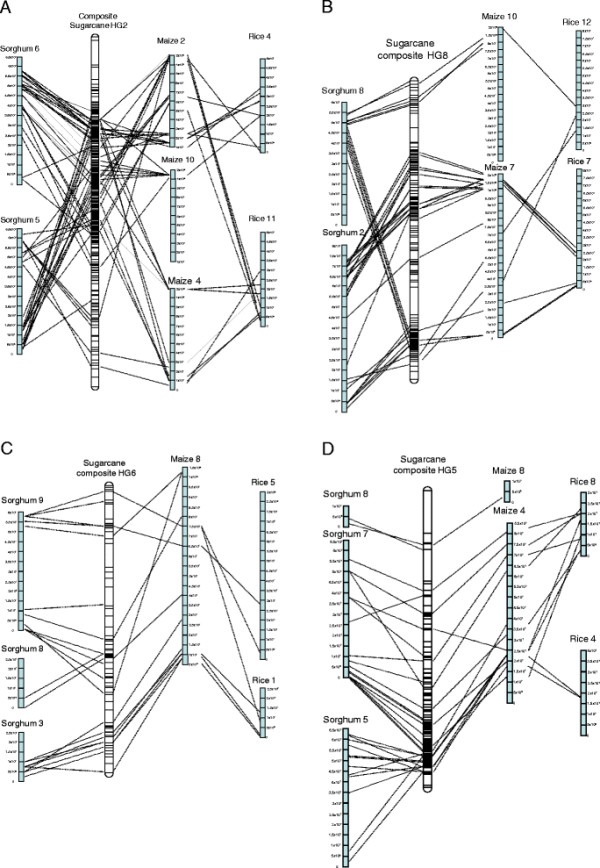
**Alignment of the composite sugarcane LGs to sorghum, maize and rice genomic sequences (A-D).** The bars on the sugarcane LG represent markers from Additional file 1. These are aligned using the BLASTN algorithm (P < e^**−**20^) and the position indicated by lines to the other chromosomes. The scale of the chromosomes is in bp.

Four major rearrangements were identified from syntenic analysis between the sugarcane genetic map and the sorghum genome (Figure 2A-D). Two of these account for the reduction in basic chromosome number from x = 10 in sorghum and *S. officinarum* to x = 8 in *S. spontaneum*. The complicated nature of the hybrid sugarcane genome means that only a few of the LGs within a HG have the translocation, for example in HG2, 7 of the LGs display the translocation, the other LGs align only to one of the two chromosomes (Additional file 1). This recombination results in the two sorghum chromosomes combining into one sugarcane HG. This translocation event reduces the basic chromosome number of sugarcane (Figure 2A). The other translocation event is in HG8 which aligns to Sb8 and Sb2 and again reduces the chromosome number to its final basic chromosome number of x = 8 (Figure 2B). The other major rearrangements had no effect on basic chromosome number but involved rearrangements between chromosomes. HG6 aligns in the main to Sb9 but one LG contains a significant translocation from Sb3 covering approximately 50% of Sb3 (Table 3, Figure 2C) The last rearrangement is in HG5 which aligns to Sb7 and Sb5 (Figure 2D); this appears to be a simple translocation between Sb7 and half of Sb5 and is present in 5 LGs within this HG (Additional file 1).

### Analysis of the DArT sequences

**Figure 3:**
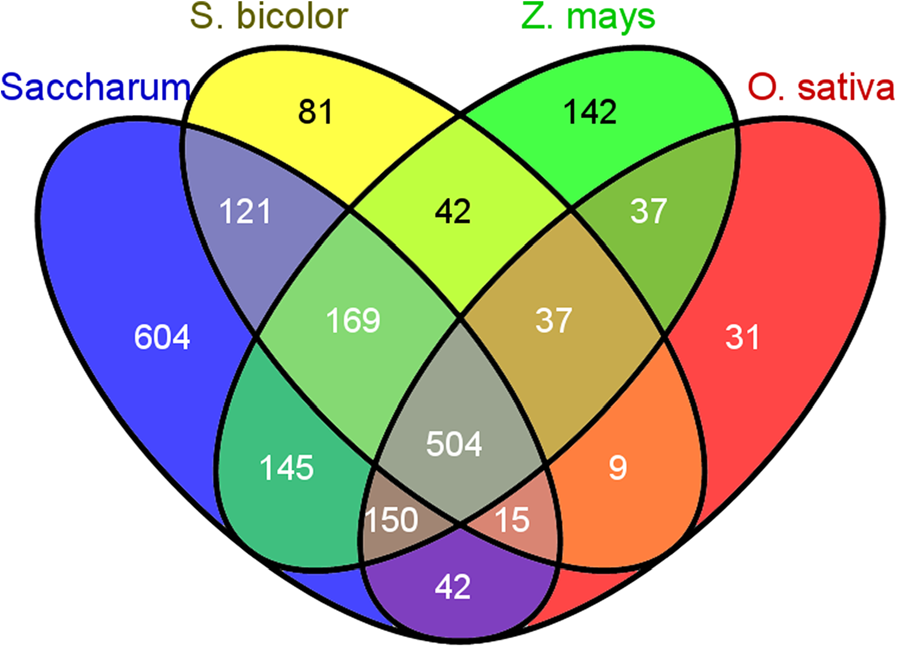
Venn diagram of DArTs with hits against the EST datasets from four species.

In total, 7846 DArT clones were sequenced and a proportion of these were also used to generate the genetic map. These sequences produced a total sequence length of 3,602,170 bp with an average length of 465 bp. These DArT sequences were assembled into a total of 5278 sequences, comprised of 1501 contigs and 3777 singletons. Of these 5278 unique sequences, 1750 hit against the *Saccharum* EST dataset, 978 to the *S. bicolor* EST dataset, 1226 to the *Z. mays* EST dataset and 825 to the *O. sativa* EST dataset. A Venn diagram representing the assembled DArT sequences that aligned to each of the relevant datasets is displayed in Figure 3. Table 4 contains the number of ESTs in each dataset from the different genomes and the total number of assembled DArT sequences that aligned to each dataset.

**Table 4:** Number of ESTs and total number of DArT sequences aligning for each EST dataset

**EST dataset species of origin**	**Number of ESTs in NCBI dataset**	**Number of assembled DArT sequences aligned**
***Saccharum***	283,254	1,750
***Sorghum bicolor***	209,828	978
***Zea mays***	2,019,114	1,226
***Oryza sativa***	1,252,989	825

The DArT markers aligned to all ten sorghum chromosomes with the largest number having homology to Sb1 (Table 5). Of the 7846 DArT markers 976 were mapped into the Q165 linkage map. All HGs contained DArT markers, 772 were from unique sequences which resulted in 18% redundancy of the mapped markers. Of the mapped DArT markers 34% had hits to genes (Table 5).

**Table 5:** Total number of DArT markers mapped in sugarcane and aligned to each sorghum chromosome using the BLASTN algorithm

**Sorghum bicolour chromosomes**	**Total number of DArT markers**	**Total number of DArT markers on the map**	**Total no. of unique DArT markers on the Q165 map**	**Hits to NCBI of mapped DArTs**	**Number of mapped DArT markers that are not genes**	**Number with no hit to NCBI**	**Percent of DArT markers mapped that are genes**
Sb1	553	128	105	23^b^ (32)^a^	62 (76)	20	27
Sb2 and Sb8	426 (Sb2) 250 (Sb8)	134	113	33 (45)	56 (65)	24	37
Sb3	449	75	61	20 (28)	38 (44)	3	34
Sb4	395	126	99	38 (54)	52 (63)	9	42
Sb5 and Sb6	247 (Sb5) 307 (Sb6)	198	154	28 (30)	100 (142)	26	22
Sb7	252	94	82	24 (27)	47 (56)	11	34
Sb9	252	107	86	27 (40)	51 (59)	8	35
Sb10	320	89	72	23 (37)	35 (38)	14	40
No hits	1783						
Super contigs	44						
Total	5278	951	772	216	441	115	34

^a^total number of individual DArT markers.

^b^total number of unique markers.

### Genome sequence comparison

**Figure 4:**
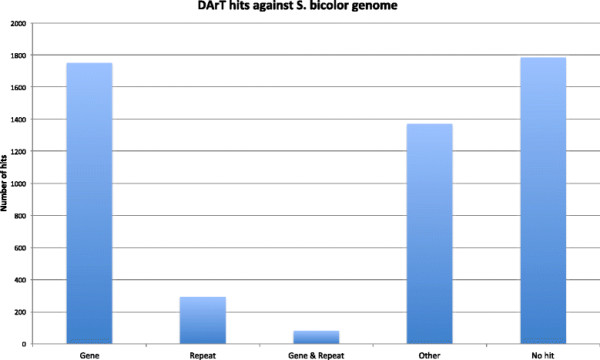
**Annotations of assembled DArT sequence alignments to different parts of the annotated S** ***.*** **bicolor genome including genes, repeats, the boundary between a repeat and genes and other which are regions that are not annotated.**

**Figure 5:**
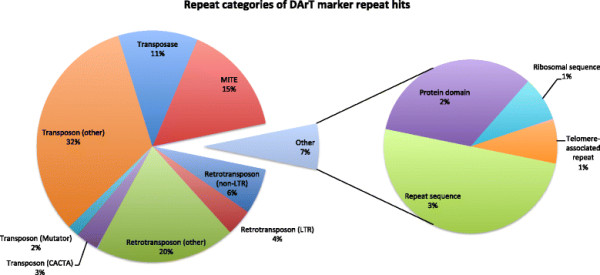
**Categories of repeats in the** ***S. bicolor*** **genome against which assembled DArT sequences aligned.**

A results table of the DArT sequence comparison against the genomes of *S. bicolor*, *Z. mays*, and *O. sativa* is provided in the Additional file 2. A total of 1755 assembled DArT sequences aligned to an annotated gene in the *S. bicolor* genome, while a similar number (1783) did not align to the genome at all. A small proportion of the DArT sequences aligned to repeats (Figure 4). The categories of repeats against which the DArT sequences aligned on the *S. bicolor* genome are displayed in Figure 5.

**Figure 6:**
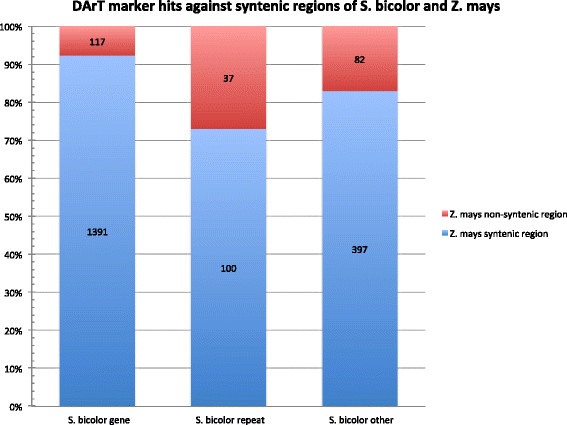
**Ratio of DArT sequences aligning in** ***S. bicolor*** **and** ***Z. mays*** **syntenic to non-syntenic regions.**

**Figure 7:**
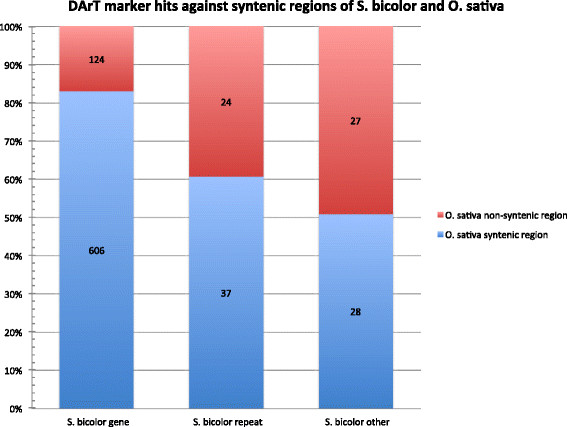
**Ratio of DArT sequences aligning in** ***S. bicolor*** **and** ***O. sativa*** **syntenic to non-syntenic regions.**

In total, 2124 assembled DArT sequences which hit against the *S. bicolor* genome sequence also hit against the *Z. mays* genome sequence, of which 1888 (88.9%) aligned to regions syntenic between these two species [[31]]. In contrast, only 846 assembled DArT sequences hitting against the *S. bicolor* genome also hit against the *O. sativa* genome sequence, of which only 671 (79.3%) aligned in regions syntenic between these two species. The ratios of syntenic to non-syntenic region alignments for each category of annotation are displayed in Figures 6 and 7. In the case of both *Z. mays* and *O. sativa*, DArT sequences aligning to annotated genes most frequently aligned to syntenic regions (92.2% and 83.0% respectively).

## Discussion

### Comparison of the sugarcane genetic map to the sorghum genome

To date only low resolution maps have been used to compare the sugarcane genetic map to the sorghum genome [[12],[32],[33]]. These maps were constructed with low-copy RFLP markers that were selected for their ability to provide a signal in cross-hybridisation thereby limiting the ability to assess orthologous and paralogous relationships in gene families, since comparative mapping by RFLP often identifies paralogous rather than orthologous sequences [[29]]. The large number of markers with known sequence in this study made it possible for the first time to align a significant part of the sugarcane genetic map to the sorghum genome sequence (Table 3, Additional file 1). Although with 954 sequences this analysis is at the macro level and only the major structural changes are identified, at the micro level there could be many more rearrangements but more markers and sequence information is needed to verify these. To use as many markers as possible in the comparison to sorghum a composite linkage map was generated where possible for each HG. This was then aligned to the sorghum genome sequence (Figure 1). Six HGs aligned to one sorghum chromosome and four HGs aligned in the main to two sorghum chromosomes. For all chromosomes there were regions that appeared to contain markers from other chromosomes (Additional file 1, Table 2) but to be considered for further analysis a number of linked markers from the same chromosomes showing homology to gene sequences had to be identified. This left four regions that showed strong likelihood of being due to rearrangement. A major translocation was identified in HG8, where LG3 aligned to Sb8 and Sb2. This result agrees with previous attempts to align sugarcane to sorghum [[32],[33]] although the greater number of markers in this map means a higher level of resolution of the alignment. Two sugarcane populations were studied by [[12]] and they determined that no more than four or five major interchromosomal translocations had occurred since the *Saccharum*-Sorghum divergence. They also identified a translocation between Sb2 and Sb8 in both *S. spontaneum* parents and the hybrid parents of their populations. They identified additional possible translocations between sorghum chromosomes 8 and 7 in the hybrid parent and 6 and 7 and 2 and 4 in the *S. spontaneum* parent, these were not verified in this study. They did not identify the Sb5/Sb6 translocation that was identified in this analysis and by [[32],[33]]. These two major translocations resulted in both cases in the condensation of two chromosomes into one. This process is known in many lineages and is demonstrated within the grass family where all grasses have evolved from an ancestral genome of 12 chromosomes [[29],[34]]. A further 2 HGs contained translocated chromosomes although these did not result in reduced chromosome number. HG5 aligned in the main to Sb7 but 5 of the 13 LGs also aligned to Sb5 indicating a translocation between Sb7 and Sb5. An interchromosomal rearrangement between LGs that aligned to Sb7 and Sb5 was also identified by [[12]] in the hybrid parent of the cross studied. The additional interchromosomal rearrangement in HG6 LG20 which aligns to Sb9 and Sb3 has not previously been identified (Table 3, Additional file 1).

### Translocations

A more detailed comparison of the four major interchromosomal rearrangements (Figure 2A-D) revealed that in some cases the rearrangement of the grass ancestral genome that has occurred in maize is the same as in sugarcane [[29]]. The chromosomal rearrangement seen in HG6 on LG20, which aligns to Sb9 and part of Sb3, corresponds to maize chromosome 8 (Figure 2C). Maize diverged from sorghum approximately 12 million years ago (mya) followed by allopolyploidisation approximately 5 mya [[29]]. The present maize chromosomes are a result of numerous rearrangements which reduced the chromosome number from 20 to 10 [[29]]. Recent work by [[35]] has shown that the whole genome duplication (WGD) which occurred in the ancestor of both maize and sorghum 29 mya has resulted in large duplicated blocks in their genomes. Sb9 of sorghum shares a duplicated block with Sb3 these two chromosomes have recombined to form maize m8. The same rearrangement has occurred in sugarcane as seen in LG20 in HG6 (Figure 2C). The translocation identified in HG2 which aligns to Sb5 and Sb6 is also seen in the top of maize m2 (Figure 2A). Parts of HG2 also align to m10 and m4 which are the duplicated ancestral regions [[26]]. The rearrangement in HG5 which aligns to Sb7 and Sb5 again is present in maize and aligns to m4 (Figure 2D). HG8 aligns to sorghum Sb8 and Sb2 which also aligns to maize m1 and m7. Interestingly the ancestral fusion event in Sb2 is retained in both maize and sugarcane although a further rearrangement of this chromosome has occurred uniquely in sugarcane (Figure 2C).

### Saccharum spontaneum

Sugarcane being a polyploid, is more tolerant of gene deletions and rearrangements than a diploid. It would appear that the rearrangements in HG2 and HG8 are major chromosome rearrangements to account for the smaller number of basic chromosomes in *S. spontaneum*. A sparse RFLP map was generated by [[10]] of a *S. spontaneum* clone SES208 using 234 single dose RFLP markers. From comparative mapping of the RFLP clones to sorghum and maize, HG4 of this map contains clones UMC130 and UMC102 which align to Sb8 and SG298 and UMC139 which align to Sb2 and are on the same LG. This is comparable to the Q165 HG8 which aligns to Sb8 and Sb2. Similarly RFLP markers in HG2 of SES208 align to Sb5 and Sb6 as does the Q165 HG2. The translocations in HG2 and HG8 appear to be inherited from the *S. spontaneum* part of the genome of Q165 but with the current data it is not possible to distinguish *S. spontaneum* chromosome from *S. officinarum* chromosomes. However, as *S. officinarum* is x = 10, the assumption is that at least the two rearrangements in HG2 and HG8 are inherited from the *S. spontaneum* part of the genome. The other rearrangements in HG5 and HG6 could be inherited from either or both species.

### Comparison within the Poaceae

Recently a detailed genetic map was constructed for another member of the Andropogoneae: Poaceae, *Miscanthus sinensis*. This species has a diploid inheritance patterns (2 n = 38) and when aligned to sorghum contains two sub-genomes, each syntenic to the entire sorghum genome with one major structural rearrangement which resulted in the condensation of two chromosomes into one and a reduction in chromosome number to x = 19 [[36]]. In this case the translocated LG aligned to Sb7 and Sb4 a translocation not seen in *Saccharum* but again seen in maize on m4. Interestingly [[36]] found that the *M. sinensis* LGs that were syntenic to Sb5 had the lowest percentage of markers that mapped to sorghum, a similar finding to switchgrass. The data suggests that linkages syntenic to Sb5 have encountered more evolutionary changes than other chromosomes after the species diverged from each other. Similarly HG2 in *Sacharrum* which is syntenic to Sb5 has undergone more rearrangement than other HGs.

**Figure 8:**
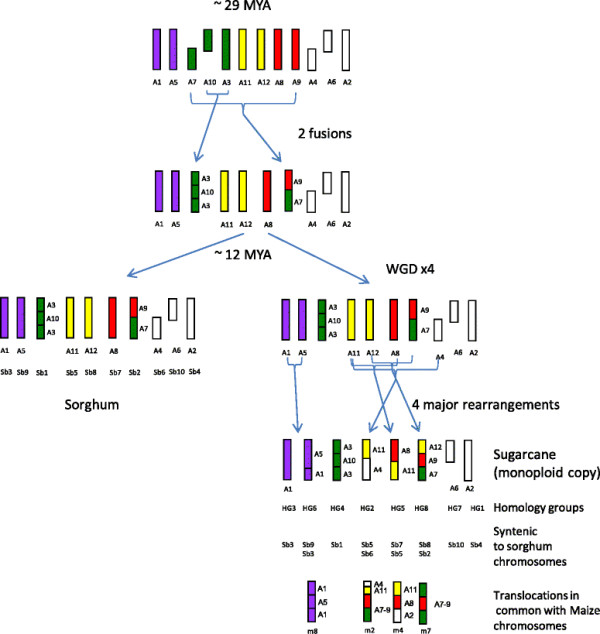
**Model for the structural evolution of the sugarcane monoploid genome (modified from[**[26]**]).** As in [[26]] the ancestral chromosomes are colour coded to represent the original 5 chromosomes (and labelled A1-12) which after a whole genome duplication (~90 MYA) and breakage/fusion formed the 12 ancestral chromosomes of sorghum.

Recent work by [[37]] comparing sequenced genomes of grasses has demonstrated that the chromosome number variation/reduction from the n = 12 common paleo-ancestor to the n = 10 of sorghum could be due to the insertion of a chromosome in the centromeric region of another chromosome although these reductions could also come about through pericentric inversions followed by reciprocal translocations [[38]]. The comparison of the Q165 *Saccharum* genetic map to the sorghum and maize genomes demonstrates *Saccharum* contains the same two ancestral fusion events. Moreover, out of the 4 major translocations identified, 3 of them have also occurred in maize (Figures 2 and 8), one of which resulted in a reduction in chromosome number. *S. officinarum* is an octoploid and *S. spontaneum* ranges from 2n = 5x = 40 to 16x = 128 [[39],[40]] in chromosome number. It would seem probable that after the whole genome duplication that also occurred in maize the *Saccharum* ancestor underwent some of the same rearrangements as maize then diverged and under went further WGD events. More sequence analysis is needed to determine how these events occurred but in evolutionary terms they would have been recent (less than 5 mya).

Whole genome duplication and the rapid and massive structural changes which have occurred in the *Poaceae* and to a greater extent in the *Saccharum* might explain the ability of these polyploids to adapt and survive environmental conditions that are not tolerated by their diploid ancestors. Within the *Saccharum* there are no known diploids as all species are polyploid. *S. spontaneum* has the widest distribution from Japan and Indonesia through the Indian subcontinent to the Mediterranean and Africa [[39]] and the largest range of ploidy levels (2n = 40-128). It has been reported that polyploidy provides increased vigour [[41]] and provided an advantage for colonizing a wider range of environments [[42]].

### Dart sequence comparison to EST and genome sequences

It is to be expected that the highest number of DArTs would align to ESTs from *Saccharum* as the genome of origin. Interestingly, a higher number of DArTs align to the maize EST dataset (1226) than to the sorghum (978) EST dataset, even though sorghum is generally thought to be a much closer relative to sugarcane [[25]] (Figure 3). This may be attributable to the higher volume of repetitive DNA in the maize genome [[25],[43]], some of which may be expressed and represented in the EST database but missing from the sorghum EST database which is much smaller than the maize database. As would be expected, the least number of DArT sequences aligned to the rice EST dataset, which is the most distant relative of the species surveyed.

A proportionally low number of DArT sequences aligned to annotated repeats (7.7%) in the sorghum genome compared to the number of DArTs aligned to annotated genes (35.1%), given that 55% of the sorghum genome is known to be repetitive and the repetitive content of sugarcane has been demonstrated to be similar to that of sorghum [[25],[44]]. This suggests that the enrichment for unique regions applied in the production of the DArT markers has largely succeeded. Interestingly, 1783 (33.8%) of the DArT marker sequences can be observed in Figure 2 to display no significant alignment to the sorghum genome, suggesting that these originate from divergent regions of sugarcane.

## Conclusion

Both the analysis of the sequence data and the comparative mapping has indicated that sugarcane is closely related to both maize and sorghum. Three out of the four translocations identified from the sugarcane genetic map are also present in the maize genome (Figure 8). These events occurred in maize after a WGD which also occurred in the ancestors of sugarcane indicating its possible sugarcane diverged after the initial rearrangements occurred. This study has highlighted the need for generation of a denser genetic map and ultimately the sugarcane genome sequence to allow the determination of the evolution of these three closely related species, sorghum, maize and sugarcane.

## Methods

### Plant material

The mapping population used to generate the Q165 genetic map consisted of 227 progeny derived from a cross between a *S. officinarum* clone IJ76-514 (2n = 80) as the female parent and Q165 (2n = 115) an Australian sugarcane cultivar and elite parent as the male parent.

### Sequencing of the DArT clones

A total of 7846 DArT clones, the majority of which were present on the DArT array used to genotype the mapping populations were isolated and DNA purified using QIAGEN plasmid purification kit. Each of these clones were sequenced using the ABI PRISM BigDye Terminator Cycle Sequencing Ready reaction kit (Perkin-Elmer, Wellesley, MA) and analyzed on an ABI 3700 sequencer. Sequencing was done with both M13 Forward and Reverse primers. The sequences were analyzed using the Lasergene software program Seqman (DNAstar Inc., Madison, WI). The vector and adapter sequences were removed and the original restriction sites restored. The DArT sequences can be obtained on request from the corresponding author.

### DArT sequence clustering and analysis

The 7846 raw DArT sequences were assembled using Sequencher with a minimum overlap length of 50 nucleotides. The EST sequences available in the NCBI EST database as at 27th October 2011 were downloaded to produce four species-specific EST datasets for *Saccharum*, *Sorghum bicolor*, *Zea mays*, and *Oryza sativa*. For each DArT contig, coding DNA sequences (CDS) were predicted by comparing the DArT sequence to these databases using TBLASTX [[45]] with an e-value cutoff of ≤10^−20^, and the best hit for each DArT sequence identified by e-value.

### Comparison with public genome sequences

Genome sequences for *S. bicolor*, *Z. mays* and *O. sativa* as well as the gene and RepeatMasker annotations for *S. bicolor* were downloaded from Phytozome version 7.0 [[46]]. The 5278 assembled DArT sequences were compared to each genome sequence using BLASTN [[40]] with an e-value cutoff of ≤10^−20^, identifying the best hit for each DArT sequence along with the chromosomal location of these hits.

We compared the position of each top DArT marker hit on the *S. bicolor* genome sequence against the annotations for genes and repeats using a custom Python script to identify whether a DArT sequence could be ‘annotated’ as one of four categories; aligned to an annotated gene, aligned to an annotated repeat, aligned on the boundary between an annotated repeat and an annotated gene, or in a region without an annotation. Assembled DArT sequences without a BLAST hit against the *S. bicolor* genome were also recorded and DArT hits against repeats were categorised.

We then compared the position of hits against the *S. bicolor* genome with the position of hits against the genomes of *Z. mays*, and *O. sativa* to identify whether DArT sequences hit against syntenic regions in these genomes based on the syntenic regions identified by [[47]].

### Marker analysis and genotyping

The DNA extraction and marker data were generated as in [[15]]. This consisted of the original data from [[15]] to which were added further AFLP, SSR, RFLP, SNP and DArT markers. Generation of further SSR, and *Pst* I/*Mse* I AFLP marker data were as reported by [[15]]. The SNP data was generated as in [[48]] and RFLP data as in [[49]]. The DArT data was generated as in [[23]]. The genetic map was produced using 2283 simplex markers that were present as a single copy in Q165, the map is reported in detail in [[24]]. Composite LGs were generated using JoinMap V4 [[50]].

### Comparison to the sorghum genome

Sequence information from the DArT, SNP and EST - SSR markers were used to align the Q165 linkage map to the genome sequence of *Sorghum bicolour*. This was carried out using BLASTN homology search; sequences had to show at least 80% nucleotide identity for at least 50% of the marker sequence length with an e-value cutoff of ≤10^−20^ to be used for comparative analysis. The results were visualised using Sigmaplot. This high stringency was used as it is difficult to infer orthologous (derived from a common ancestor by speciation) and paralogous (derived by duplication within one genome) relationships from sequence comparisons using a less stringent e-value.

## Competing interests

Andrzej Kilian is Director of Diversity Arrays Technology who provide DArT array commercial genotyping services for a range of crops.

## Authors’ contributions

KSA carried out the comparative mapping analysis and drafted the manuscript. SH, JL, AK, PCB and MDM generated the molecular data. PJB conducted the sequence analysis and KA, MDM, PCB and PJB critically revised the manuscript. All authors read and approved the final manuscript.

## Additional files

## Supplementary Material

Additional file 1: Figure 1 Linkage map of the sugarcane cultivar Q165 aligned to the sorghum genome (Sb numbers are the assigned chromosomes names). The linkage groups (LGs) were placed into Homology Groups (HGs) firstly using allelic information from the SSR, SNP and RFLP markers. Secondly the location of the LG within a HG was confirmed with sequence information from 677 markers with known sequence that has a primary correspondence to the sorghum genome at a significance level of P <e^−20^. Coloured text represents markers with homology to the sorghum genome at P <e^−20^. The texts in boxes are alleles that are present more than once in the HG and used to assign LGs to HGs. The grey text are markers with homology at P <e^−20^ to other sorghum chromosomes. The black text is markers with no sequence information or no hit to the sorghum genome. In total 958 points of comparison between sugarcane and sorghum are used for the comparison. Alleles of markers are linked by dotted lines. DArT markers that form contigs and group on the map are surrounded by brackets. (Details of map in [44]). Click here for file

Additional file 2 **A full results table of the DArT sequence comparison against the genomes of** ***S. bicolour*,** ***Z. mays*** **and** ***O. sativa.***
Click here for file
