# Supplementary material for: Comparative mapping in the Poaceae family reveals translocations in the complex polyploid genome of sugarcane
Source: BMC Plant Biol. 2014 Jul 26;14:190. doi: 10.1186/s12870-014-0190-x (PMC4222257; doi:10.1186/s12870-014-0190-x)
Supplement: Additional file 1: Figure 1 — Linkage map of the sugarcane cultivar Q165 aligned to the sorghum genome (Sb numbers are the assigned chromosomes names). The linkage groups (LGs) were placed into Homology Groups (HGs) firstly using allelic information from the SSR, SNP and RFLP markers. Secondly the location of the LG within a HG was confirmed with sequence information from 677 markers with known sequence that has a primary correspondence to the sorghum genome at a significance level of P <e−20. Coloured text represents markers with homology to the sorghum genome at P <e−20. The texts in boxes are alleles that are present more than once in the HG and used to assign LGs to HGs. The grey text are markers with homology at P <e−20 to other sorghum chromosomes. The black text is markers with no sequence information or no hit to the sorghum genome. In total 958 points of comparison between sugarcane and sorghum are used for the comparison. Alleles of markers are linked by dotted lines. DArT markers that form contigs and group on the map are surrounded by brackets. (Details of map in [44]). [file s12870-014-0190-x-S1.ppt]

## Slide 1
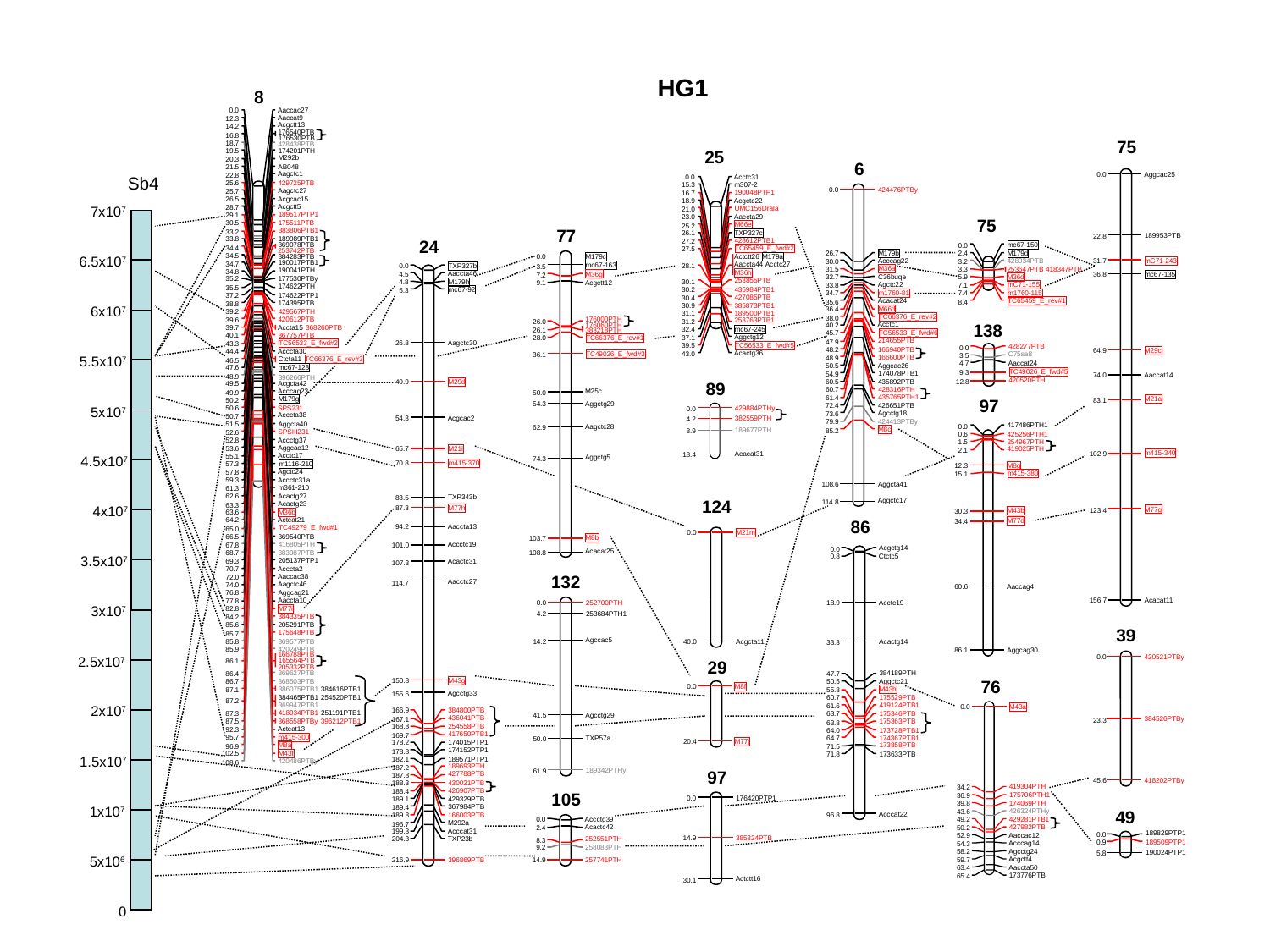

HG1
8
0.0
Aaccac27
Aaccat9
12.3
Acgctt13
14.2
176540PTB
16.8
176530PTB
75
0.0
Aggcac25
189953PTB
22.8
31.7
mC71-243
36.8
mc67-135
64.9
M29c
74.0
Aaccat14
M21a
83.1
m415-340
102.9
M77g
123.4
156.7
Acacat11
18.7
428438PTB
19.5
174201PTH
25
0.0
Acctc31
15.3
m307-2
190048PTP1
16.7
18.9
Acgctc22
UMC156DraIa
21.0
23.0
Aaccta29
M66e
25.2
26.1
TXP327c
428612PTB1
27.2
TC65459_E_fwd#2
27.5
Actctt26
M179a
Aaccta44
Acctc27
28.1
M36h
253855PTB
30.1
30.2
435984PTB1
427085PTB
30.4
30.9
385873PTB1
31.1
189500PTB1
253763PTB1
31.2
32.4
mc67-245
Aggctg12
37.1
39.5
TC56533_E_fwd#5
Acactg36
43.0
M292b
20.3
6
0.0
424476PTBy
26.7
M179b
Acccag22
30.0
M36a
31.5
32.7
C36buqe
Agctc22
33.8
34.7
m1760-81
Acacat24
35.6
36.4
M66d
TC66376_E_rev#2
38.0
Acctc1
40.2
45.7
TC56533_E_fwd#6
214655PTB
47.9
48.2
166940PTB
166600PTB
48.9
50.5
Aggcac26
174078PTB1
54.9
60.5
435892PTB
60.7
428316PTH
435765PTH1
61.4
72.4
426651PTB
Agcctg18
73.6
79.9
424413PTBy
M8c
85.2
108.6
Aggcta41
Aggctc17
114.8
21.5
AB048
Sb4
Aagctc1
22.8
25.6
429725PTB
Aagctc27
25.7
26.5
Acgcac15
7x10^7^
Acgctt5
28.7
189517PTP1
29.1
75
mc67-150
0.0
2.4
M179d
428034PTB
3.2
3.3
253647PTB
418347PTB
5.9
M36d
mC71-155
7.1
7.4
m1760-115
TC65459_E_rev#1
8.4
30.5
175511PTB
77
0.0
M179c
mc67-163
3.5
M36g
7.2
9.1
Acgctt12
176000PTH
26.0
176060PTH
26.1
383218PTH
28.0
TC66376_E_rev#1
TC49026_E_fwd#3
36.1
M25c
50.0
54.3
Aggctg29
Aagctc28
62.9
Aggctg5
74.3
M8b
103.7
Acacat25
108.8
383806PTB1
33.2
33.8
189989PTB1
24
0.0
TXP327b
Aaccta46
4.5
4.8
M179h
mc67-92
5.3
26.8
Aagctc30
40.9
M29d
54.3
Acgcac2
65.7
M21i
70.8
m415-370
TXP343b
83.5
87.3
M77h
94.2
Aaccta13
Accctc19
101.0
Acactc31
107.3
Aacctc27
114.7
150.8
M43g
Agcctg33
155.6
166.9
384800PTB
436041PTB
167.1
168.8
254558PTB
417650PTB1
169.7
178.2
174015PTP1
174152PTP1
178.8
182.1
189571PTP1
189693PTH
187.2
427788PTB
187.8
188.3
430021PTB
426907PTB
188.4
189.1
429329PTB
367984PTB
189.4
189.8
166003PTB
M292a
196.7
199.3
Acccat31
204.3
TXP23b
216.9
396869PTB
369078PTB
34.4
6.5x10^7^
253742PTB
34.5
384283PTB
190017PTB1
34.7
190041PTH
34.8
35.2
177530PTBy
174622PTH
35.5
37.2
174622PTP1
6x10^7^
174395PTB
38.8
39.2
429567PTH
420612PTB
39.6
138
428277PTB
0.0
C75sa8
3.5
4.7
Aaccat24
TC49026_E_fwd#5
9.3
420520PTH
12.8
39.7
Accta15
368260PTB
40.1
367757PTB
TC56533_E_fwd#2
43.3
5.5x10^7^
44.4
Acccta30
Ctcta11
TC66376_E_rev#3
46.5
47.6
mc67-128
48.9
396266PTH
89
429884PTHy
0.0
382559PTH
4.2
189677PTH
8.9
Acacat31
18.4
49.5
Acgcta42
Acccag23
49.9
M179g
97
417486PTH1
0.0
0.6
425256PTH1
1.5
254967PTH
419025PTH
2.1
12.3
M8g
m415-380
15.1
M43b
30.3
M77d
34.4
60.6
Aaccag4
86.1
Aggcag30
50.2
5x10^7^
50.6
SPS231
Acccta38
50.7
51.5
Aggcta40
SPSIII231
52.6
52.8
Accctg37
Aggcac12
53.6
4.5x10^7^
Acctc17
55.1
57.3
m1116-210
Agctc24
57.8
59.3
Accctc31a
m361-210
61.3
62.6
Acactg27
124
0.0
M21m
40.0
Acgcta11
4x10^7^
Acactg23
63.3
63.6
M36b
64.2
Actcat21
86
Acgctg14
0.0
0.8
Ctctc5
18.9
Acctc19
Acactg14
33.3
384189PTH
47.7
50.5
Aggctc21
M43h
55.8
60.7
175529PTB
419124PTB1
61.6
63.7
175346PTB
175363PTB
63.8
64.0
173728PTB1
64.7
174367PTB1
173858PTB
71.5
71.8
173633PTB
Acccat22
96.8
TC49279_E_fwd#1
65.0
66.5
369540PTB
416805PTH
67.8
3.5x10^7^
68.7
383987PTB
205137PTP1
69.3
70.7
Acccta2
132
0.0
252700PTH
4.2
253684PTH1
Agccac5
14.2
41.5
Agcctg29
TXP57a
50.0
189342PTHy
61.9
Aaccac38
72.0
Aagctc46
74.0
76.8
Aggcag21
Aaccta10
3x10^7^
77.8
82.8
M77i
384335PTB
84.2
85.6
205291PTB
39
0.0
420521PTBy
384526PTBy
23.3
45.6
418202PTBy
175648PTB
85.7
85.8
369577PTB
85.9
420249PTB
2.5x10^7^
166788PTB
29
0.0
M8f
20.4
M77j
165564PTB
86.1
205332PTB
369627PTB
86.4
76
M43a
0.0
419304PTH
34.2
175706PTH1
36.9
174069PTH
39.8
426324PTHy
43.6
429281PTB1
49.2
427982PTB
50.2
Aaccac12
52.9
Acccag14
54.3
Agcctg24
58.2
Acgctt4
59.7
Aaccta50
63.4
173776PTB
65.4
86.7
368503PTB
386075PTB1
384616PTB1
87.1
384465PTB1
254520PTB1
2x10^7^
87.2
369947PTB1
418934PTB1
251191PTB1
87.3
87.5
368558PTBy
396212PTB1
Actcat13
92.3
95.7
m415-300
M8a
96.9
1.5x10^7^
102.5
M43f
420486PTBy
108.6
97
0.0
176420PTP1
14.9
385324PTB
Actctt16
30.1
105
0.0
Accctg39
Acactc42
2.4
252551PTH
8.3
9.2
258083PTH
14.9
257741PTH
1x10^7^
49
189829PTP1
0.0
0.9
189509PTP1
190024PTP1
5.8
5x10^6^
0

## Slide 2
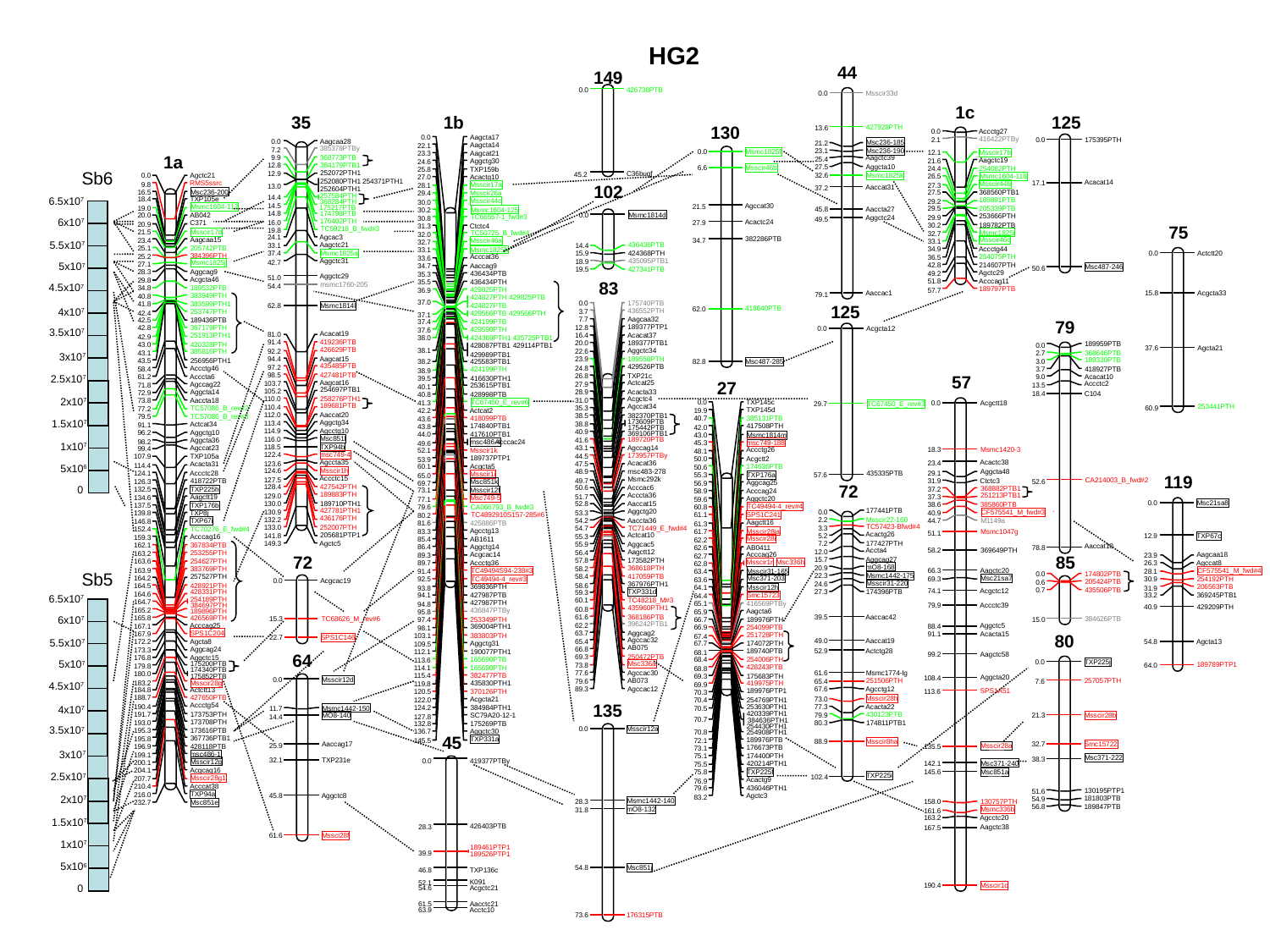

HG2
44
0.0
Msscir33d
427928PTH
13.6
Msc236-185
21.2
23.1
Msc236-190
Aagctc39
25.4
27.5
Aggcta10
32.6
Msmc1825k
Aaccat31
37.2
45.8
Aaccta27
Aggctc24
49.5
Aaccac1
79.1
149
0.0
426738PTB
C36buqf
45.2
1c
0.0
Accctg27
416422PTBy
2.1
12.1
Msscir17b
Aagctc19
21.6
24.4
254082PTH
26.5
Msmc1604-118
Msscir44b
27.3
27.5
368560PTB1
189891PTB
29.2
29.5
205339PTB
253666PTH
29.9
30.2
189782PTB
Msmc1825i
32.7
Msscir46c
33.1
34.9
Accctg44
254075PTH
36.5
42.8
214607PTH
Agctc29
49.2
51.8
Acccag11
189797PTB
57.7
35
1b
0.0
Aagcta17
Aagcta14
22.1
23.3
Aagcat21
Aggctg30
24.6
25.8
TXP159b
27.0
Acactg10
Msscir17a
28.1
29.4
Msscir26a
Msscir44c
30.0
30.2
Msmc1604-125
TC66557-1_fwd#3
30.8
31.3
Ctctc4
TC50725_B_fwd#4
32.0
Msscir46a
32.7
33.1
Msmc1825h
Acccat36
33.6
34.7
Aaccag9
436434PTB
35.3
35.5
436434PTH
429825PTH
36.9
424827PTH
429825PTB
37.0
424827PTB
429566PTB
429566PTH
37.1
37.4
424199PTB
429590PTH
37.6
38.0
424369PTH1
435725PTB1
428087PTB1
429114PTB1
38.1
429989PTB1
38.2
425583PTB1
424199PTH
38.9
39.5
416630PTH1
253615PTB1
40.1
40.8
428998PTB
TC67450_E_rev#6
41.3
42.2
Actcat2
418099PTB
43.6
174840PTB1
43.8
44.0
417610PTB1
msc486-4
Acccac24
49.6
52.1
Msscir1k
189737PTP1
53.9
60.1
Acgcta5
Msscir1i
65.0
Msc851k
69.7
73.1
Msscir12f
Msc749-5
77.1
79.6
CA066793_B_fwd#3
TC4892910S157-285#6
80.2
81.6
425886PTB
Agcctg13
83.3
85.4
AB1611
86.4
Aggctg14
Acgcac14
89.3
89.7
Accctg36
TC49494S94-238#3
91.4
92.5
TC49494-4_rev#3
369836PTH
93.8
94.1
427987PTB
427987PTH
94.8
436847PTBy
95.8
97.4
253349PTH
369004PTH1
98.1
103.1
383803PTH
Aggctg31
109.5
112.1
190077PTH1
165690PTB
113.6
114.1
165690PTH
115.4
382477PTB
435830PTH1
119.8
120.5
370126PTH
Acgcta21
122.0
124.2
384984PTH1
SC79A20-12-1
127.8
132.8
175269PTB
136.7
Aggctc30
TXP331a
145.5
125
0.0
175395PTH
Acacat14
17.1
Msc487-246
50.6
130
0.0
Msmc1825f
6.6
Msscir46b
Agccat30
21.5
Acactc24
27.9
382286PTB
34.7
418640PTB
62.0
82.8
Msc487-285
0.0
Aagcaa28
385378PTBy
7.2
1a
9.9
368773PTB
Sb6
12.8
384179PTB1
252072PTH1
12.9
0.0
Agctc21
252080PTH1
254371PTH1
RMS5ssrc
9.8
102
0.0
Msmc1814d
436436PTB
14.4
15.9
424368PTH
435095PTB1
18.9
19.5
427341PTB
13.0
252604PTH1
16.5
Msc236-200
6.5x10^7^
257584PTH
14.4
18.4
TXP105e
368284PTH
14.5
Msmc1604-112
175217PTB
19.0
6x10^7^
14.8
174798PTB
20.0
AB042
176402PTH
16.0
C371
20.9
75
0.0
Actctt20
15.8
Acgcta33
37.6
Agcta21
253441PTH
60.9
TC59218_B_fwd#3
19.8
21.5
Msscir17d
5.5x10^7^
24.1
Agcac3
Aagcaa15
23.4
Aagctc21
33.1
25.1
205742PTB
37.4
Msmc1825a
384396PTH
25.2
5x10^7^
Aggctc31
42.7
Msmc1825j
27.1
28.3
Aggcag9
Aggctc29
51.0
4.5x10^7^
Acgcta46
29.8
83
msmc1760-205
54.4
34.8
189532PTB
383949PTH
40.8
4x10^7^
0.0
175740PTB
41.8
383599PTH1
125
0.0
Acgcta12
29.7
TC67450_E_rev#3
435335PTB
57.6
62.8
Msmc1814l
436552PTH
253747PTH
3.7
42.4
7.7
Aagcaa32
79
189959PTB
0.0
2.7
368646PTB
189330PTB
3.0
3.7
418927PTB
9.0
Acacat10
Accctc2
13.5
18.4
C104
CA214003_B_fwd#2
52.6
Aaccat10
78.8
42.5
189436PTB
3.5x10^7^
189377PTP1
42.8
367179PTH
12.8
Acacat19
81.0
251913PTH1
16.4
Acacat37
42.9
91.4
419236PTB
20.0
189377PTB1
43.0
420328PTH
3x10^7^
426629PTB
Aggctc34
92.2
385816PTH
22.6
43.1
94.4
Aagcat15
23.9
189558PTH
43.5
256956PTH1
435485PTB
429526PTB
97.2
Accctg46
24.8
58.4
2.5x10^7^
98.5
427481PTB
26.8
TXP21c
57
0.0
Acgctt18
18.3
Msmc1420-3
Acactc38
23.4
Aggcta48
29.1
31.9
Ctctc3
368882PTB1
37.2
251213PTB1
37.3
38.6
385860PTB
CF575541_M_fwd#3
40.9
44.7
M1149a
Msmc1047g
51.1
58.2
369649PTH
66.3
Aagctc20
Msc21sa7
69.3
74.1
Acgctc12
79.9
Accctc39
Aggctc5
88.4
91.1
Acacta15
99.2
Aagctc58
Aggcta20
108.4
SPS1A51
113.6
Msscir28a
135.5
142.1
Msc371-240
145.6
Msc851a
158.0
130757PTH
Msmc336b
161.6
163.2
Agcctc20
Aagctc38
167.5
190.4
Msscir1c
61.2
Acccta6
27
Aagcat16
Actcat25
103.7
Agccag22
27.9
71.8
254697PTB1
105.2
Aggcta14
28.9
Acacta33
2x10^7^
72.9
110.0
258276PTH1
Acgctc4
73.8
Aaccta18
31.0
0.0
TXP145c
189681PTB
Agccat34
110.4
TC57086_B_rev#2
35.3
77.2
TXP145d
19.9
112.0
Aaccat20
1.5x10^7^
38.5
382370PTB1
79.5
TC57086_B_rev#3
40.7
385131PTB
173609PTB
Aggctg34
113.4
Actcat34
38.8
91.1
417508PTH
42.0
175442PTB
114.9
Agcctg10
40.9
96.2
Aggctg10
369106PTB1
43.0
Msmc1814m
1x10^7^
Msc851l
189720PTB
116.0
Aggcta36
41.6
98.2
45.3
msc749-188
118.5
TXP94b
Agccat23
43.1
Agccag14
99.4
Accctg26
48.1
122.4
msc749-4
173957PTBy
107.9
TXP105a
44.5
50.0
Acgctt2
5x10^6^
Agccta35
Acacat36
123.6
Acacta31
47.5
114.4
174636PTB
50.6
124.6
Msscir1h
48.9
msc483-278
124.1
Accctc28
55.3
TXP176a
119
0.0
Msc21sa8
12.9
TXP67c
Aagcaa18
23.9
26.3
Agccat8
CF575541_M_fwd#4
28.1
30.9
254192PTH
206563PTB
31.9
33.2
369245PTB1
40.9
429209PTH
54.8
Agcta13
189789PTP1
64.0
Accctc15
Msmc292k
127.5
418722PTB
49.7
0
126.3
Aggcag25
56.9
72
177441PTB
0.0
2.2
Msscir22-160
TC57423-Bfwd#4
3.3
Acactg26
5.2
7.2
177427PTH
Accta4
12.0
15.7
Aggcag27
mO8-168
20.9
22.3
Msmc1442-175
Msscir31-220
24.6
27.3
174396PTB
39.5
Aaccac42
49.0
Aaccat19
52.9
Actctg28
61.6
Msmc1774-lg
251506PTH
65.4
67.6
Agcctg12
Msscir28h
73.0
77.3
Acacta22
430123PTB
79.9
80.3
174811PTB1
88.9
Msscir8ha
TXP225i
102.4
128.4
427542PTH
50.6
Acccac6
132.5
TXP225h
58.9
Acccag24
189883PTH
Acccta36
129.0
Aagctt19
51.7
134.6
Aggctc20
59.6
130.0
189710PTH1
52.8
Aaccat15
137.5
TXP176b
TC49494-4_rev#4
60.8
427781PTH1
Aggctg20
130.9
139.8
TXP8j
53.3
61.1
SPS1C241
436176PTH
132.2
TXP67i
54.2
Aaccta36
146.8
Aagctt16
61.3
133.0
252007PTH
54.7
TC71449_E_fwd#4
152.4
TC70276_E_fwd#4
61.7
Msscir28ia
205681PTP1
Actcat10
141.8
Acccag16
55.3
159.3
Msscir28i
62.2
149.3
Agctc5
55.9
Aggcac5
162.1
367834PTB
62.6
AB0411
Aagctt12
253255PTH
56.4
163.2
Acccag26
72
0.0
Acgcac19
15.3
TC68626_M_rev#6
22.7
SPS1C146
85
0.0
174802PTB
205424PTB
0.6
0.7
435506PTB
384626PTB
15.0
62.7
57.8
173582PTH
163.6
254627PTH
Msscir1r
Msc336h
62.8
Sb5
368618PTH
383769PTH
58.2
163.9
63.4
Msscir31-165
257527PTH
58.4
417059PTB
164.2
Msc371-203
63.6
367976PTH1
164.5
428921PTH
58.6
64.1
Msscir12h
6.5x10^7^
428331PTH
TXP331d
59.3
164.6
Smc15723
64.4
254189PTH
60.1
TC48218_M#3
164.7
65.1
416569PTBy
384697PTH
435960PTH1
60.8
165.2
Aagcta6
189896PTH
6x10^7^
65.9
61.6
368186PTB
165.8
426569PTH
66.7
189976PTH
396242PTB1
Acccag25
62.2
167.1
66.9
254099PTB
SPS1C204
63.7
Aggcag2
167.9
5.5x10^7^
80
0.0
TXP225j
257057PTH
7.6
21.3
Msscir28b
32.7
Smc15722
Msc371-222
38.3
130195PTP1
51.6
181803PTB
54.9
56.8
189847PTB
251728PTH
67.4
Agccac32
172.2
Agcta8
65.4
67.7
174072PTH
AB075
Aggcag24
66.8
173.3
189740PTB
68.1
64
0.0
Msscir12d
11.7
Msmc1442-150
MO8-140
14.4
Aaccag17
25.9
32.1
TXP231e
45.8
Aggctc8
61.6
Mssci28f
5x10^7^
69.3
250472PTB
176.8
Aggctc15
68.4
254006PTH
175200PTB
Msc336a
73.8
179.8
428243PTB
68.8
174340PTB
77.6
Agccac30
180.0
175852PTB
69.3
175683PTH
4.5x10^7^
AB073
79.6
183.2
Msscir28g
419975PTH
69.9
89.3
Agccac12
184.8
Actctt13
189976PTP1
70.3
188.7
427650PTB
70.4
254769PTH1
4x10^7^
135
0.0
Msscir12a
Msmc1442-140
28.3
mO8-132
31.8
54.8
Msc851j
73.6
176315PTB
Accctg54
190.4
253630PTH1
70.5
420339PTH1
191.7
173753PTH
70.7
384636PTH1
173708PTH
3.5x10^7^
193.0
254430PTH1
195.3
173616PTB
70.8
254908PTH1
45
0.0
419377PTBy
426403PTB
28.3
189461PTP1
39.9
189526PTP1
46.8
TXP136c
K091
52.1
54.6
Acgctc21
61.5
Aacctc21
63.9
Acctc10
367736PTB1
195.8
189976PTB
72.1
3x10^7^
196.9
428118PTB
176673PTB
73.1
msc486-1
199.1
75.1
174400PTH
Msscir12g
200.1
420214PTH1
75.5
2.5x10^7^
204.1
Acgcag16
75.8
TXP225f
Msscir28g1
207.7
Acactg9
76.9
210.4
Acccat38
79.6
436046PTH1
2x10^7^
TXP94a
216.0
Agctc3
83.2
232.7
Msc851e
1.5x10^7^
1x10^7^
5x10^6^
0

## Slide 3
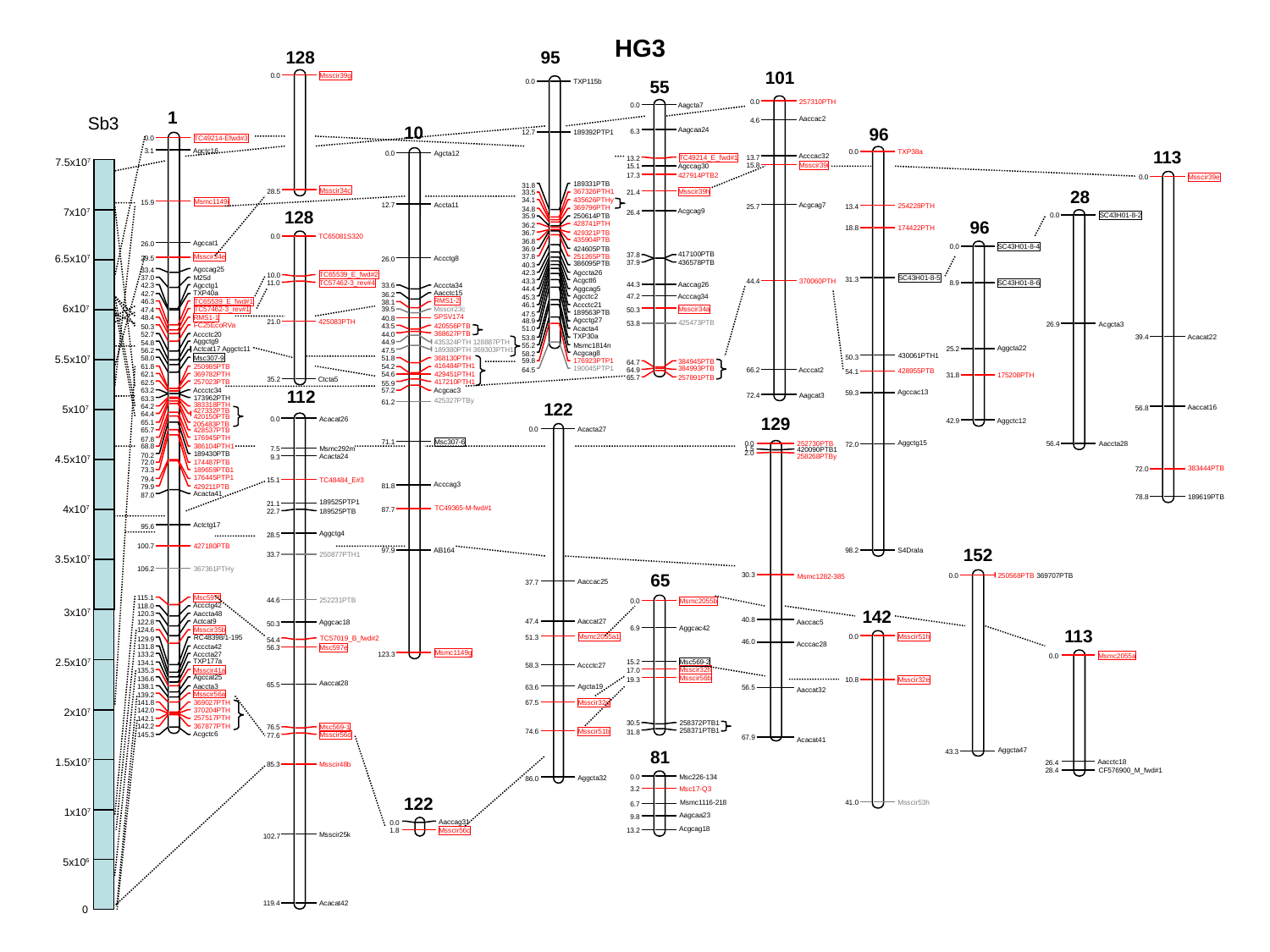

HG3
128
0.0
Msscir39g
Msscir34c
28.5
95
0.0
TXP115b
12.7
189392PTP1
189331PTB
31.8
367326PTH1
33.5
34.1
435626PTHy
369796PTH
34.8
35.9
250614PTB
428741PTH
36.2
36.7
429321PTB
435904PTB
36.8
36.9
424605PTB
37.8
251265PTB
386095PTB
40.3
42.3
Agccta26
Acgctt6
43.3
44.4
Aggcag5
Agcctc2
45.3
46.1
Accctc21
189563PTB
47.5
Agcctg27
48.9
51.0
Acacta4
TXP30a
53.8
55.2
Msmc1814n
Acgcag8
58.2
59.8
176923PTP1
190045PTP1
64.5
101
0.0
257310PTH
Aaccac2
4.6
Acccac32
13.7
15.8
Msscir39i
Acgcag7
25.7
44.4
370060PTH
66.2
Acccat2
72.4
Aagcat3
55
0.0
Aagcta7
Aagcaa24
6.3
TC49214_E_fwd#1
13.2
15.1
Agccag30
17.3
427914PTB2
Msscir39h
21.4
Acgcag9
26.4
417100PTB
37.8
37.9
436578PTB
44.3
Aaccag26
47.2
Acccag34
Msscir34a
50.3
425473PTB
53.8
384945PTB
64.7
384993PTB
64.9
65.7
257891PTB
Sb3
1
10
0.0
Agcta12
12.7
Accta11
Accctg8
26.0
33.6
Acccta34
Aacctc15
36.2
RMS1-2
38.1
39.5
Msscir23c
SPSV174
40.8
43.5
420556PTB
368627PTB
44.0
44.9
435324PTH
128887PTH
189380PTH
369303PTH1
47.5
51.8
368130PTH
416484PTH1
54.2
54.6
429451PTH1
417210PTH1
55.9
57.2
Acgcac3
425327PTBy
61.2
71.1
Msc307-6
Acccag3
81.8
TC49365-M-fwd#1
87.7
97.9
AB164
Msmc1149g
123.3
96
0.0
TXP38a
254228PTH
13.4
18.8
174422PTH
SC43H01-8-5
31.3
430061PTH1
50.3
428955PTB
54.1
Agccac13
59.3
Aggctg15
72.0
98.2
S4DraIa
0.0
TC49214-Efwd#3
3.1
Agctc16
113
0.0
Msscir39e
39.4
Acacat22
Aaccat16
56.8
383444PTB
72.0
78.8
189619PTB
7.5x10^7^
28
0.0
SC43H01-8-2
26.9
Acgcta3
56.4
Aaccta28
Msmc1149i
15.9
7x10^7^
128
0.0
TC65081S320
TC65539_E_fwd#2
10.0
11.0
TC57462-3_rev#4
21.0
425083PTH
35.2
Ctcta5
96
0.0
SC43H01-8-4
8.9
SC43H01-8-6
Aggcta22
25.2
31.8
175208PTH
42.9
Aggctc12
Agccat1
26.0
6.5x10^7^
Msscir34e
29.5
Agccag25
33.4
37.0
M25d
42.3
Agcctg1
TXP40a
42.7
6x10^7^
46.3
TC65539_E_fwd#1
TC57462-3_rev#1
47.4
48.4
RMS1-1
FC25EcoRVa
50.3
52.7
Accctc20
Aggctg9
54.8
Actcat17
Aggctc11
56.2
5.5x10^7^
58.0
Msc307-9
61.8
250985PTB
62.1
369782PTH
257023PTB
62.5
112
0.0
Acacat26
7.5
Msmc292m
Acacta24
9.3
15.1
TC48484_E#3
189525PTP1
21.1
22.7
189525PTB
Aggctg4
28.5
33.7
250877PTH1
44.6
252231PTB
Aggcac18
50.3
TC57019_B_fwd#2
54.4
56.3
Msc597e
Aaccat28
65.5
76.5
Msc569-1
Msscir56d
77.6
85.3
Msscir48b
Msscir25k
102.7
119.4
Acacat42
63.2
Accctc34
173962PTH
63.3
5x10^7^
122
0.0
Acacta27
Aaccac25
37.7
47.4
Aaccat27
Msmc2055a1
51.3
58.3
Accctc27
Agcta19
63.6
67.5
Msscir32g
74.6
Msscir51b
Aggcta32
86.0
383318PTH
64.2
427332PTB
64.4
420150PTB
129
0.0
252730PTB
1.5
420090PTB1
2.0
258268PTBy
30.3
Msmc1282-385
40.8
Aaccac5
46.0
Acccac28
56.5
Aaccat32
67.9
Acacat41
65.1
205483PTB
65.7
428537PTB
176945PTH
67.8
68.8
386104PTH1
4.5x10^7^
189430PTB
70.2
72.0
174487PTB
73.3
189659PTB1
176445PTP1
79.4
79.9
429211PTB
Acacta41
87.0
4x10^7^
Actctg17
95.6
100.7
427180PTB
152
0.0
250568PTB
369707PTB
Aggcta47
43.3
3.5x10^7^
106.2
367361PTHy
65
0.0
Msmc2055b
6.9
Aggcac42
15.2
Msc569-2
Msscir32h
17.0
Msscir56b
19.3
30.5
258372PTB1
258371PTB1
31.8
115.1
Msc597f
3x10^7^
Accctg42
118.0
142
0.0
Msscir51h
10.8
Msscir32e
41.0
Msscir53h
120.3
Aaccta48
Actcat9
122.8
113
0.0
Msmc2055a
Aacctc18
26.4
28.4
CF576900_M_fwd#1
124.6
Msscir35b
RC48398/1-195
129.9
131.8
Acccta42
2.5x10^7^
133.2
Acccta27
TXP177a
134.1
135.3
Msscir41a
Agccat25
136.6
138.1
Aaccta3
Msscir56a
139.2
141.8
369027PTH
2x10^7^
142.0
370204PTH
257517PTH
142.1
142.2
367877PTH
Acgctc6
145.3
81
0.0
Msc226-134
3.2
Msc17-Q3
Msmc1116-218
6.7
Aagcaa23
9.8
Acgcag18
13.2
1.5x10^7^
122
Aaccag31
0.0
1.8
Msscir56c
1x10^7^
5x10^6^
0

## Slide 4
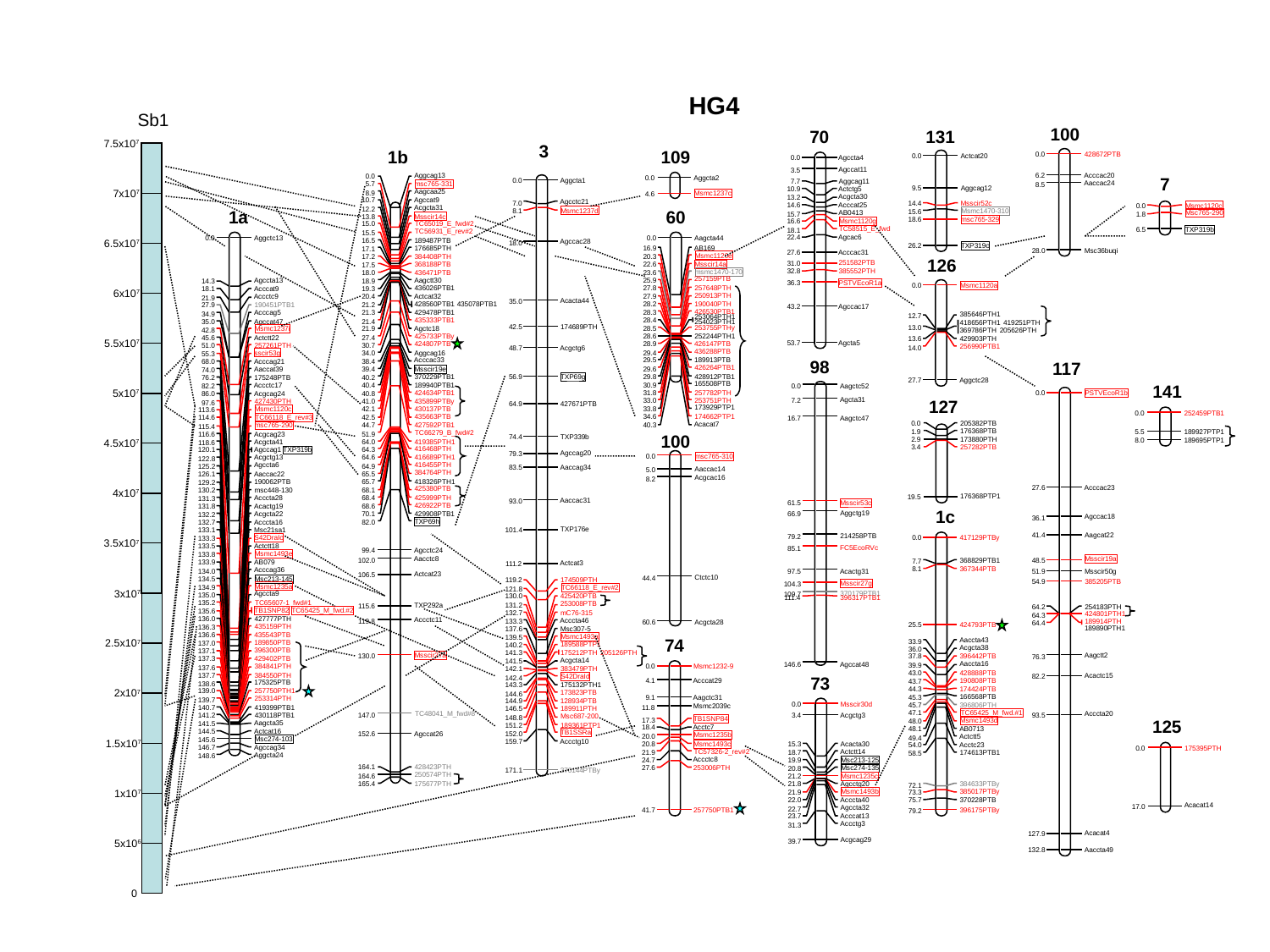

HG4
Sb1
100
0.0
428672PTB
6.2
Acccac20
Aaccac24
8.5
28.0
Msc36buqi
70
0.0
Agccta4
Agccat11
3.5
7.7
Aggcag11
10.9
Actctg5
Acgcta30
13.2
14.6
Acccat25
AB0413
15.7
16.6
Msmc1120g
TC58515_E_fwd
18.1
22.4
Agcac6
27.6
Acccac31
251582PTB
31.0
32.8
385552PTH
36.3
PSTVEcoR1a
43.2
Agccac17
53.7
Agcta5
131
0.0
Actcat20
9.5
Aggcag12
14.4
Msscir52c
Msmc1470-310
15.6
18.6
msc765-329
26.2
TXP319c
7.5x10^7^
3
0.0
Aggcta1
Agcctc21
7.0
8.1
Msmc1237d
Agccac28
18.0
Acacta44
35.0
42.5
174689PTH
48.7
Acgctg6
56.9
TXP69g
64.9
427671PTB
74.4
TXP339b
Agccag20
79.3
83.5
Aaccag34
Aaccac31
93.0
TXP176e
101.4
Actcat3
111.2
119.2
174509PTH
TC66118_E_rev#2
121.8
130.0
425420PTB
253008PTB
131.2
132.7
mC76-315
Acccta46
133.3
137.6
Msc307-5
Msmc1493a
139.5
189588PTP1
140.2
141.3
175212PTH
205126PTH
Acgcta14
141.5
142.1
383479PTH
S42DraId
142.4
143.3
175132PTH1
173823PTB
144.6
144.9
128934PTB
146.5
189911PTH
Msc687-200
148.8
151.2
189361PTP1
TB1SSRa
152.0
159.7
Accctg10
171.1
370144PTBy
1b
Aggcag13
0.0
5.7
msc765-331
Aagcaa25
8.9
10.7
Agccat9
Acgcta31
12.2
13.8
Msscir14c
15.0
TC65019_E_fwd#2
TC56931_E_rev#2
15.5
16.5
189487PTB
176685PTH
17.1
17.2
384408PTH
368188PTB
17.5
18.0
436471PTB
Aagctt30
18.9
436026PTB1
19.3
20.4
Actcat32
428560PTB1
435078PTB1
21.2
21.3
429478PTB1
435333PTB1
21.4
21.9
Agctc18
425733PTBy
27.4
424807PTB
30.7
34.0
Aggcag16
Acccac33
38.4
39.4
Msscir19e
370229PTB1
40.2
40.4
189940PTB1
424634PTB1
40.8
41.0
435899PTBy
42.1
430137PTB
435663PTB
42.5
44.7
427592PTB1
TC66279_B_fwd#2
51.9
64.0
419385PTH1
416468PTH
64.3
64.6
416689PTH1
416455PTH
64.9
384764PTH
65.5
65.7
418326PTH1
425380PTB
68.1
68.4
425999PTH
426922PTB
68.6
70.1
429908PTB1
TXP69h
82.0
99.4
Agcctc24
Aacctc8
102.0
Actcat23
106.5
TXP292a
115.6
Accctc11
119.8
Msscir27a
130.0
TC48041_M_fwd#8
147.0
152.6
Agccat26
164.1
428423PTH
250574PTH
164.6
165.4
175677PTH
109
0.0
Aggcta2
Msmc1237c
4.6
7
0.0
Msmc1120c
Msc765-290
1.8
6.5
TXP319b
7x10^7^
1a
0.0
Aggctc13
Agccta13
14.3
18.1
Acccat9
Accctc9
21.9
27.9
190451PTB1
Acccag5
34.9
35.0
Agccat47
Msmc1237i
42.8
45.6
Actctt22
51.0
257261PTH
sscir53g
55.3
68.0
Acccag21
Aaccat39
74.0
76.2
175248PTB
Accctc17
82.2
86.0
Acgcag24
427430PTH
97.6
Msmc1120c
113.6
114.6
TC66118_E_rev#3
msc765-290
115.4
116.6
Acgcag23
Acgcta41
118.6
120.1
Agccag1
TXP319b
Acgctg13
122.8
Agccta6
125.2
126.1
Aaccac22
190062PTB
129.2
130.2
msc448-130
Acccta28
131.3
131.8
Acactg19
Acgcta22
132.2
132.7
Acccta16
133.1
Msc21sa1
S42DraIc
133.3
133.5
Actctt18
Msmc1493e
133.8
133.9
AB079
Acccag36
134.0
134.5
Msc213-145
Msmc1235a
134.9
Agccta9
135.0
135.2
TC65607-1_fwd#1
TB1SNP82
TC65425_M_fwd.#2
135.6
136.0
427777PTH
435159PTH
136.3
136.6
435543PTB
189850PTB
137.0
396300PTB
137.1
137.3
429402PTB
384841PTH
137.6
137.7
384550PTH
175325PTB
138.6
139.0
257750PTH1
253314PTH
139.7
140.7
419399PTB1
141.2
430118PTB1
Aagcta35
141.5
144.5
Actcat16
Msc274-103
145.6
146.7
Agccag34
Aggcta24
148.6
60
6.5x10^7^
0.0
Aagcta44
16.9
AB169
Msmc1120e
20.3
126
0.0
Msmc1120a
385646PTH1
12.7
418656PTH1
419251PTH
13.0
369786PTH
205626PTH
13.6
429903PTH
256990PTB1
14.0
27.7
Aggctc28
22.6
Msscir14a
msmc1470-170
23.6
257159PTB
25.9
6x10^7^
27.8
257648PTH
250913PTH
27.9
28.2
190040PTH
426530PTB1
28.3
253064PTH1
28.4
254023PTH1
253755PTHy
28.5
5.5x10^7^
28.6
252244PTH1
28.9
426147PTB
436288PTB
29.4
29.5
189913PTB
98
0.0
Aagctc52
Agcta31
7.2
16.7
Aagctc47
Msscir53c
61.5
Aggctg19
66.9
214258PTB
79.2
FC5EcoRVc
85.1
97.5
Acactg31
Msscir27g
104.3
370179PTB1
109.7
111.4
396317PTB1
146.6
Agccat48
117
0.0
PSTVEcoR1b
27.6
Acccac23
Agccac18
36.1
41.4
Aagcat22
Msscir19a
48.5
51.9
Msscir50g
54.9
385205PTB
64.2
254183PTH
424801PTH1
64.3
189914PTH
64.4
Aagctt2
76.3
Acactc15
82.2
Acccta20
93.5
Acacat4
127.9
132.8
Aaccta49
426264PTB1
29.6
29.8
428912PTB1
165508PTB
5x10^7^
141
0.0
252459PTB1
5.5
189927PTP1
8.0
189695PTP1
30.9
31.8
257782PTH
33.0
253751PTH
127
0.0
205382PTB
176368PTB
1.9
2.9
173880PTH
3.4
257282PTB
176368PTP1
19.5
173929PTP1
33.8
34.6
174662PTP1
Acacat7
40.3
4.5x10^7^
100
0.0
msc765-310
Aaccac14
5.0
Acgcac16
8.2
Ctctc10
44.4
60.6
Acgcta28
4x10^7^
1c
0.0
417129PTBy
368829PTB1
7.7
8.1
367344PTB
25.5
424793PTB
Aaccta43
33.9
Acgcta38
36.0
37.8
396442PTB
Aaccta16
39.9
43.0
428888PTB
190808PTB
43.7
44.3
174424PTB
166568PTB
45.3
45.7
396806PTH
47.1
TC65425_M_fwd.#1
Msmc1493d
48.0
48.1
AB0713
Actctt5
49.4
54.0
Acctc23
174613PTB1
58.5
384633PTBy
72.1
385017PTBy
73.3
75.7
370228PTB
396175PTBy
79.2
3.5x10^7^
3x10^7^
189890PTH1
2.5x10^7^
74
0.0
Msmc1232-9
4.1
Acccat29
9.1
Aagctc31
Msmc2039c
11.8
TB1SNP84
17.3
18.4
Acctc7
Msmc1235b
20.0
20.8
Msmc1493c
TC57326-2_rev#2
21.9
Accctc8
24.7
27.6
253006PTH
41.7
257750PTB1
73
0.0
Msscir30d
3.4
Acgctg3
15.3
Acacta30
Actctt14
18.7
19.9
Msc213-125
Msc274-135
20.8
21.2
Msmc1235c
21.8
Agcctg20
Msmc1493b
21.9
22.0
Acccta40
Agccta32
22.7
23.7
Acccat13
Accctg3
31.3
Acgcag29
39.7
2x10^7^
125
0.0
175395PTH
Acacat14
17.0
1.5x10^7^
1x10^7^
5x10^6^
0

## Slide 5
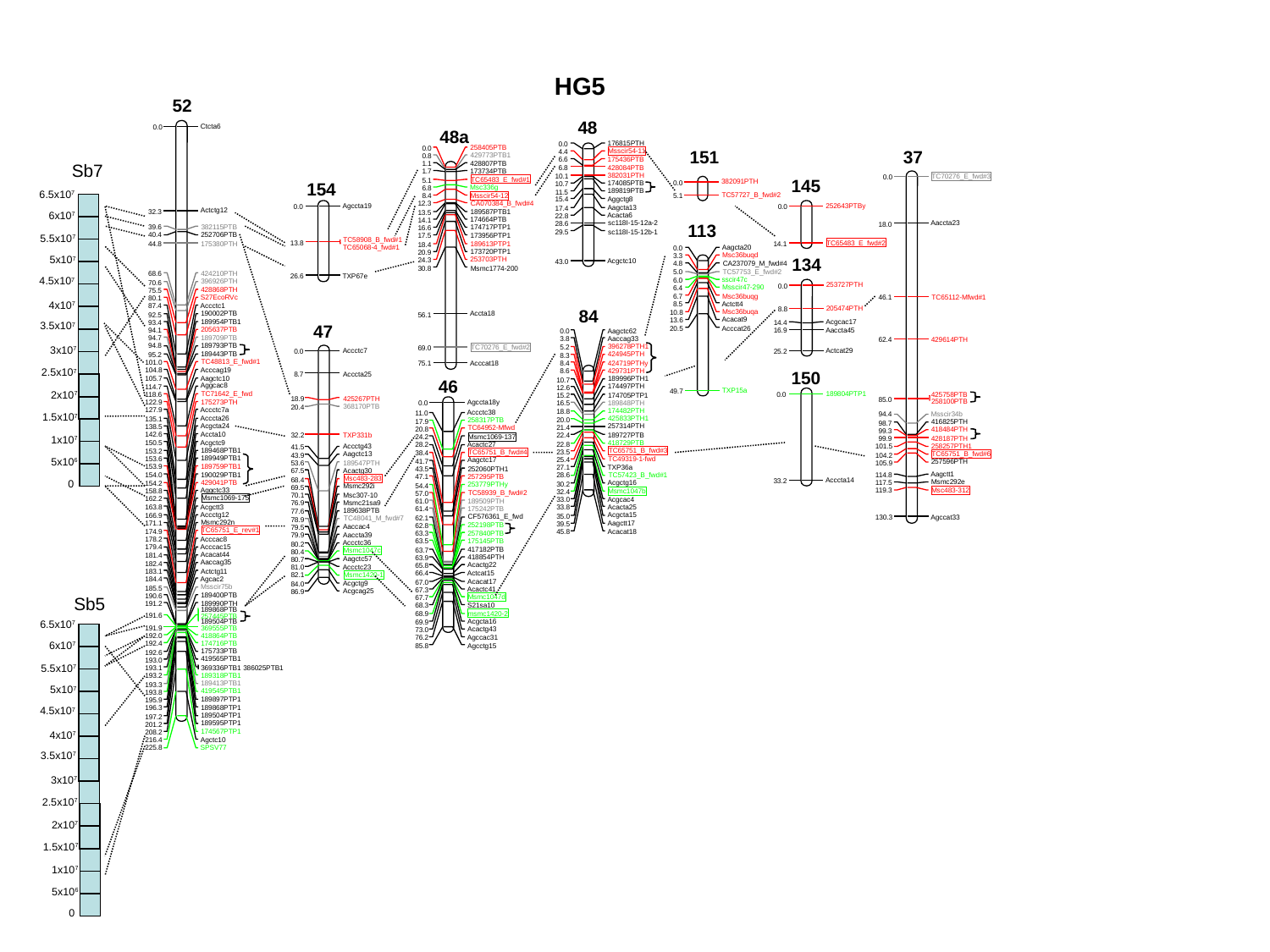

HG5
52
48
176815PTH
0.0
Msscir54-11
4.4
6.6
175436PTB
6.8
428084PTB
382031PTH
10.1
174085PTB
10.7
189819PTB
11.5
15.4
Aggctg8
Aagcta13
17.4
Acacta6
22.8
sc118I-15-12a-2
28.6
29.5
sc118I-15-12b-1
Acgctc10
43.0
Ctcta6
0.0
48a
258405PTB
0.0
429773PTB1
0.8
1.1
428807PTB
1.7
173734PTB
TC65483_E_fwd#1
5.1
Msc336g
6.8
8.4
Msscir54-12
12.3
CA070384_B_fwd#4
189587PTB1
13.5
174664PTB
14.1
174717PTP1
16.6
17.5
173956PTP1
189613PTP1
18.4
173720PTP1
20.9
253703PTH
24.3
30.8
Msmc1774-200
Accta18
56.1
TC70276_E_fwd#2
69.0
75.1
Acccat18
151
382091PTH
0.0
TC57727_B_fwd#2
5.1
37
Sb7
TC70276_E_fwd#3
0.0
145
252643PTBy
0.0
TC65483_E_fwd#2
14.1
154
Agccta19
0.0
TC58908_B_fwd#1
13.8
TC65068-4_fwd#1
26.6
TXP67e
6.5x10^7^
6x10^7^
Actctg12
32.3
Aaccta23
113
Aagcta20
0.0
Msc36buqd
3.3
4.8
CA237079_M_fwd#4
5.0
TC57753_E_fwd#2
sscir47c
6.0
Msscir47-290
6.4
6.7
Msc36buqg
8.5
Actctt4
Msc36buqa
10.8
Acacat9
13.6
20.5
Acccat26
TXP15a
49.7
18.0
39.6
382115PTB
5.5x10^7^
40.4
252706PTB
44.8
175380PTH
5x10^7^
134
253727PTH
0.0
205474PTH
8.8
Acgcac17
14.4
16.9
Aaccta45
Actcat29
25.2
4.5x10^7^
68.6
424210PTH
396926PTH
70.6
428868PTH
75.5
4x10^7^
S27EcoRVc
46.1
TC65112-Mfwd#1
80.1
87.4
Accctc1
84
0.0
Aagctc62
3.8
Aaccag33
396278PTH1
5.2
424945PTH
8.3
8.4
424719PTHy
8.6
429731PTH
189996PTH1
10.7
174497PTH
12.6
15.2
174705PTP1
16.5
189848PTH
174482PTH
18.8
425833PTH1
20.0
257314PTH
21.4
22.4
189727PTB
418729PTB
22.8
TC65751_B_fwd#3
23.5
TC49319-1-fwd
25.4
27.1
TXP36a
28.6
TC57423_B_fwd#1
Acgctg16
30.2
Msmc1047b
32.4
33.0
Acgcac4
33.8
Acacta25
Acgcta15
35.0
Aagctt17
39.5
45.8
Acacat18
190002PTB
92.5
3.5x10^7^
189954PTB1
93.4
47
Accctc7
0.0
8.7
Acccta25
18.9
425267PTH
368170PTB
20.4
32.2
TXP331b
Accctg43
41.5
Aagctc13
43.9
53.6
189547PTH
67.5
Acactg30
Msc483-283
68.4
Msmc292i
69.5
70.1
Msc307-10
76.9
Msmc21sa9
189638PTB
77.6
TC48041_M_fwd#7
78.9
Aaccac4
79.5
79.9
Aaccta39
Accctc36
80.2
Msmc1047c
80.4
Aagctc57
80.7
81.0
Accctc23
82.1
Msmc1420-1
Acgctg9
84.0
Acgcag25
86.9
205637PTB
94.1
94.7
189709PTB
62.4
429614PTH
3x10^7^
94.8
189793PTB
189443PTB
95.2
TC48813_E_fwd#1
101.0
2.5x10^7^
104.8
Acccag19
150
189804PTP1
0.0
Acccta14
33.2
105.7
Aagctc10
46
Agccta18y
0.0
Accctc38
11.0
258317PTB
17.9
TC64952-Mfwd
20.8
24.2
Msmc1069-137
28.2
Acactc27
TC65751_B_fwd#4
38.4
Aagctc17
41.7
43.5
252060PTH1
47.1
257295PTB
253779PTHy
54.4
TC58939_B_fwd#2
57.0
61.0
189509PTH
61.4
175242PTB
CF576361_E_fwd
62.1
252198PTB
62.8
63.3
257840PTB
63.5
175145PTB
417182PTB
63.7
418854PTH
63.9
Acactg22
65.8
66.4
Actcat15
Acacat17
67.0
Acactc41
67.3
Msmc1047d
67.7
68.3
S21sa10
68.9
msmc1420-2
Acgcta16
69.9
Acactg43
73.0
76.2
Agccac31
85.8
Agcctg15
Aggcac8
2x10^7^
114.7
TC71642_E_fwd
425758PTB
118.6
85.0
258100PTB
122.9
175273PTH
1.5x10^7^
127.9
Accctc7a
94.4
Msscir34b
Acccta26
135.1
416825PTH
98.7
Acgcta24
138.5
418484PTH
99.3
1x10^7^
142.6
Accta10
99.9
428187PTH
150.5
Acgctc9
101.5
258257PTH1
189468PTB1
153.2
5x10^6^
TC65751_B_fwd#6
104.2
189949PTB1
153.6
257596PTH
105.9
153.9
189759PTB1
Aagctt1
0
154.0
190029PTB1
114.8
Msmc292e
429041PTB
117.5
154.2
Aggctc33
119.3
Msc483-312
158.8
Msmc1069-175
162.2
163.8
Acgctt3
Accctg12
166.9
130.3
Agccat33
Msmc292n
171.1
TC65751_E_rev#1
174.9
178.2
Acccac8
179.4
Acccac15
Acacat44
181.4
Aaccag35
182.4
183.1
Actctg11
184.4
Agcac2
Msscir75b
185.5
Sb5
189400PTB
190.6
191.2
189990PTH
189868PTB
6.5x10^7^
191.6
257445PTB
189504PTB
191.9
369555PTB
192.0
418864PTB
6x10^7^
192.4
174716PTB
175733PTB
192.6
419565PTB1
5.5x10^7^
193.0
193.1
369336PTB1
386025PTB1
193.2
189318PTB1
5x10^7^
189413PTB1
193.3
419545PTB1
193.8
189897PTP1
195.9
4.5x10^7^
196.3
189868PTP1
189504PTP1
197.2
189595PTP1
201.2
4x10^7^
174567PTP1
208.2
216.4
Agctc10
3.5x10^7^
225.8
SPSV77
3x10^7^
2.5x10^7^
2x10^7^
1.5x10^7^
1x10^7^
5x10^6^
0

## Slide 6
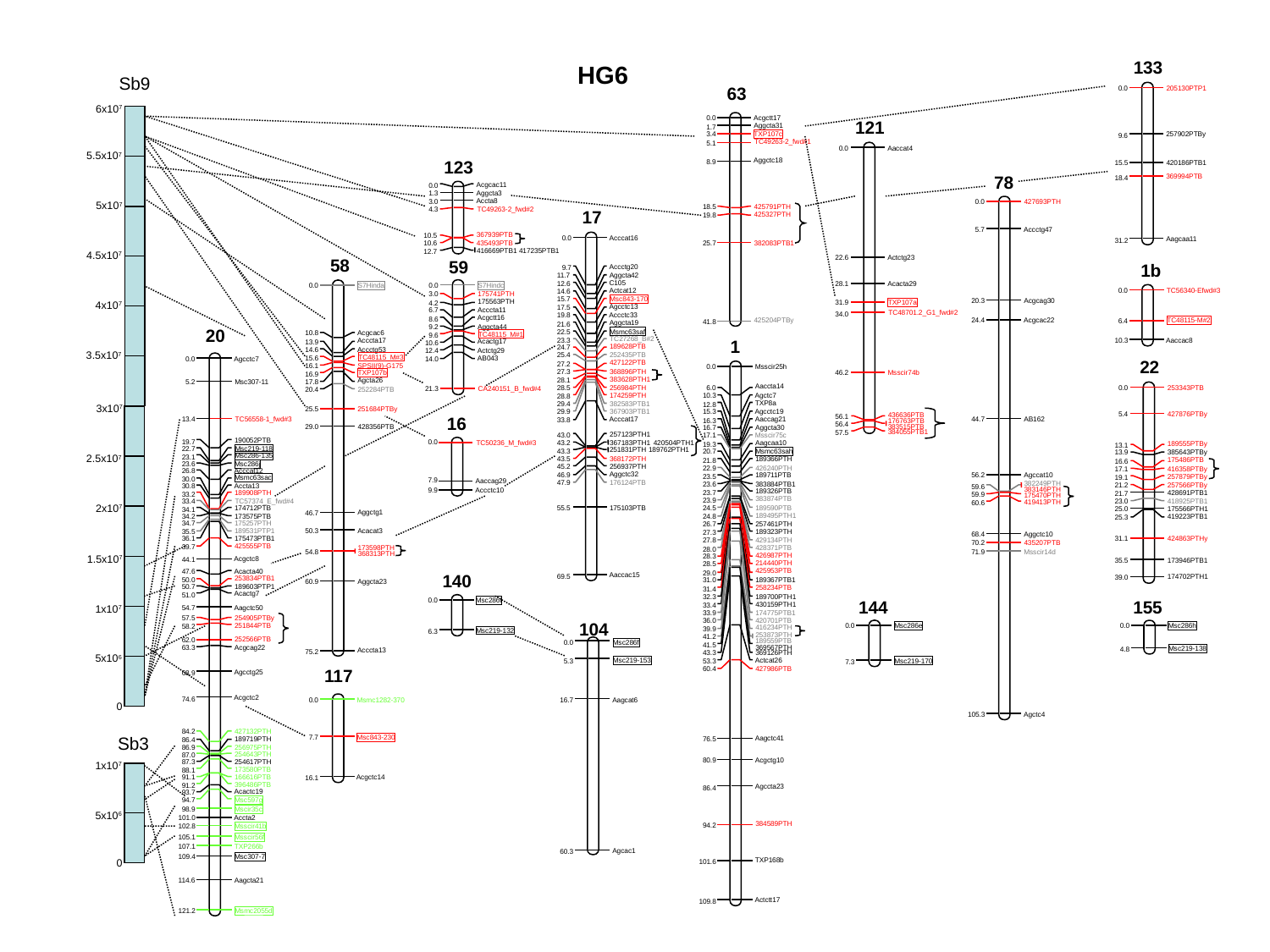

HG6
133
0.0
205130PTP1
257902PTBy
9.6
15.5
420186PTB1
369994PTB
18.4
Aagcaa11
31.2
Sb9
63
0.0
Acgctt17
Aggcta31
1.7
3.4
TXP107c
TC49263-2_fwd#1
5.1
Aggctc18
8.9
18.5
425791PTH
425327PTH
19.8
25.7
382083PTB1
425204PTBy
41.8
6x10^7^
121
5.5x10^7^
0.0
Aaccat4
123
Acgcac11
0.0
1.3
Aggcta3
Accta8
3.0
4.3
TC49263-2_fwd#2
367939PTB
10.5
10.6
435493PTB
416669PTB1
417235PTB1
12.7
78
5x10^7^
0.0
427693PTH
17
0.0
Acccat16
Accctg20
9.7
11.7
Aggcta42
C105
12.6
Actcat12
14.6
15.7
Msc843-170
Agcctc13
17.5
19.8
Accctc33
Aggcta19
21.6
22.5
Msmc63saf
TC27268_B#2
23.3
189628PTB
24.7
25.4
252435PTB
427122PTB
27.2
27.3
368896PTH
383628PTH1
28.1
28.5
256984PTH
174259PTH
28.8
29.4
382583PTB1
29.9
367903PTB1
Acccat17
33.8
257123PTH1
43.0
43.2
367183PTH1
420504PTH1
251831PTH
189762PTH1
43.3
43.5
368172PTH
45.2
256937PTH
Aggctc32
46.9
47.9
176124PTB
55.5
175103PTB
Aaccac15
69.5
5.7
Accctg47
4.5x10^7^
22.6
Actctg23
58
59
0.0
S7Hindc
3.0
175741PTH
175563PTH
4.2
6.7
Acccta11
Acgctt16
8.6
9.2
Aggcta44
TC48115_M#1
9.6
Acactg17
10.6
12.4
Actctg29
AB043
14.0
21.3
CA240151_B_fwd#4
1b
0.0
TC56340-Efwd#3
TC48115-M#2
6.4
10.3
Aaccac8
28.1
Acacta29
0.0
S7Hinda
4x10^7^
20.3
Acgcag30
31.9
TXP107a
TC48701.2_G1_fwd#2
34.0
24.4
Acgcac22
20
0.0
Agcctc7
5.2
Msc307-11
13.4
TC56558-1_fwd#3
190052PTB
19.7
22.7
Msc219-118
Msc286-135
23.1
23.6
Msc286j
26.8
Acccat12
Msmc63sac
30.0
30.8
Accta13
189908PTH
33.2
33.4
TC57374_E_fwd#4
174712PTB
34.1
34.2
173575PTB
34.7
175257PTH
189531PTP1
35.5
36.1
175473PTB1
425555PTB
39.7
Acgctc8
44.1
47.6
Acacta40
253834PTB1
50.0
50.7
189603PTP1
Acactg7
51.0
54.7
Aagctc50
57.5
254905PTBy
251844PTB
58.2
252566PTB
62.0
63.3
Acgcag22
Agcctg25
68.9
Acgctc2
74.6
84.2
427132PTH
189719PTH
86.4
86.9
256975PTH
254643PTH
87.0
87.3
254617PTH
173580PTB
88.1
91.1
166616PTB
396486PTB
91.2
Acactc19
93.7
94.7
Msc597g
98.9
Mscir35c
101.0
Accta2
102.8
Msscir41b
105.1
Msscir56f
107.1
TXP266b
109.4
Msc307-7
114.6
Aagcta21
121.2
Msmc2055d
10.8
Acgcac6
1
0.0
Msscir25h
Aaccta14
6.0
10.3
Agctc7
TXP8a
12.8
15.3
Agcctc19
Aaccag21
16.3
16.7
Aggcta30
17.1
Msscir75c
Aagcaa10
19.3
20.7
Msmc63sah
189366PTH
21.8
22.9
426240PTH
189711PTB
23.5
23.6
383884PTB1
189326PTB
23.7
383874PTB
23.9
24.5
189590PTB
189495PTH1
24.8
26.7
257461PTH
189323PTH
27.3
27.8
429134PTH
428371PTB
28.0
426987PTH
28.3
214440PTH
28.5
425953PTB
29.0
31.0
189367PTB1
258234PTB
31.4
32.3
189700PTH1
430159PTH1
33.4
33.9
174775PTB1
36.0
420701PTB
416234PTH
39.9
253873PTH
41.2
189559PTB
41.5
369567PTH
43.3
369126PTH
Actcat26
53.3
60.4
427986PTB
Aagctc41
76.5
80.9
Acgctg10
Agccta23
86.4
384589PTH
94.2
TXP168b
101.6
Actctt17
109.8
Acccta17
13.9
3.5x10^7^
14.6
Accctg53
TC48115_M#3
15.6
22
0.0
253343PTB
5.4
427876PTBy
189555PTBy
13.1
13.9
385643PTBy
175486PTB
16.6
17.1
416358PTBy
257879PTBy
19.1
21.2
257566PTBy
428691PTB1
21.7
23.0
418925PTB1
25.0
175566PTH1
419223PTB1
25.3
31.1
424863PTHy
35.5
173946PTB1
174702PTH1
39.0
16.1
SPSII(9)-G175
46.2
Msscir74b
TXP107b
16.9
Agcta26
17.8
20.4
252284PTB
3x10^7^
25.5
251684PTBy
436636PTB
56.1
16
0.0
TC50236_M_fwd#3
7.9
Aaccag29
9.9
Accctc10
44.7
AB162
176763PTB
56.4
383515PTB
29.0
428356PTB
384055PTB1
57.5
2.5x10^7^
56.2
Agccat10
382249PTH
59.6
383146PTH
59.9
175470PTH
2x10^7^
419413PTH
60.6
Aggctg1
46.7
50.3
Acacat3
68.4
Aggctc10
70.2
435207PTB
173598PTH
1.5x10^7^
54.8
71.9
Msscir14d
368313PTH
140
0.0
Msc286i
Msc219-132
6.3
60.9
Aggcta23
1x10^7^
144
0.0
Msc286e
Msc219-170
7.3
155
0.0
Msc286h
Msc219-138
4.8
104
0.0
Msc286f
Msc219-153
5.3
16.7
Aagcat6
Agcac1
60.3
5x10^6^
Acccta13
75.2
117
0.0
Msmc1282-370
7.7
Msc843-230
Acgctc14
16.1
0
105.3
Agctc4
Sb3
1x10^7^
5x10^6^
0

## Slide 7
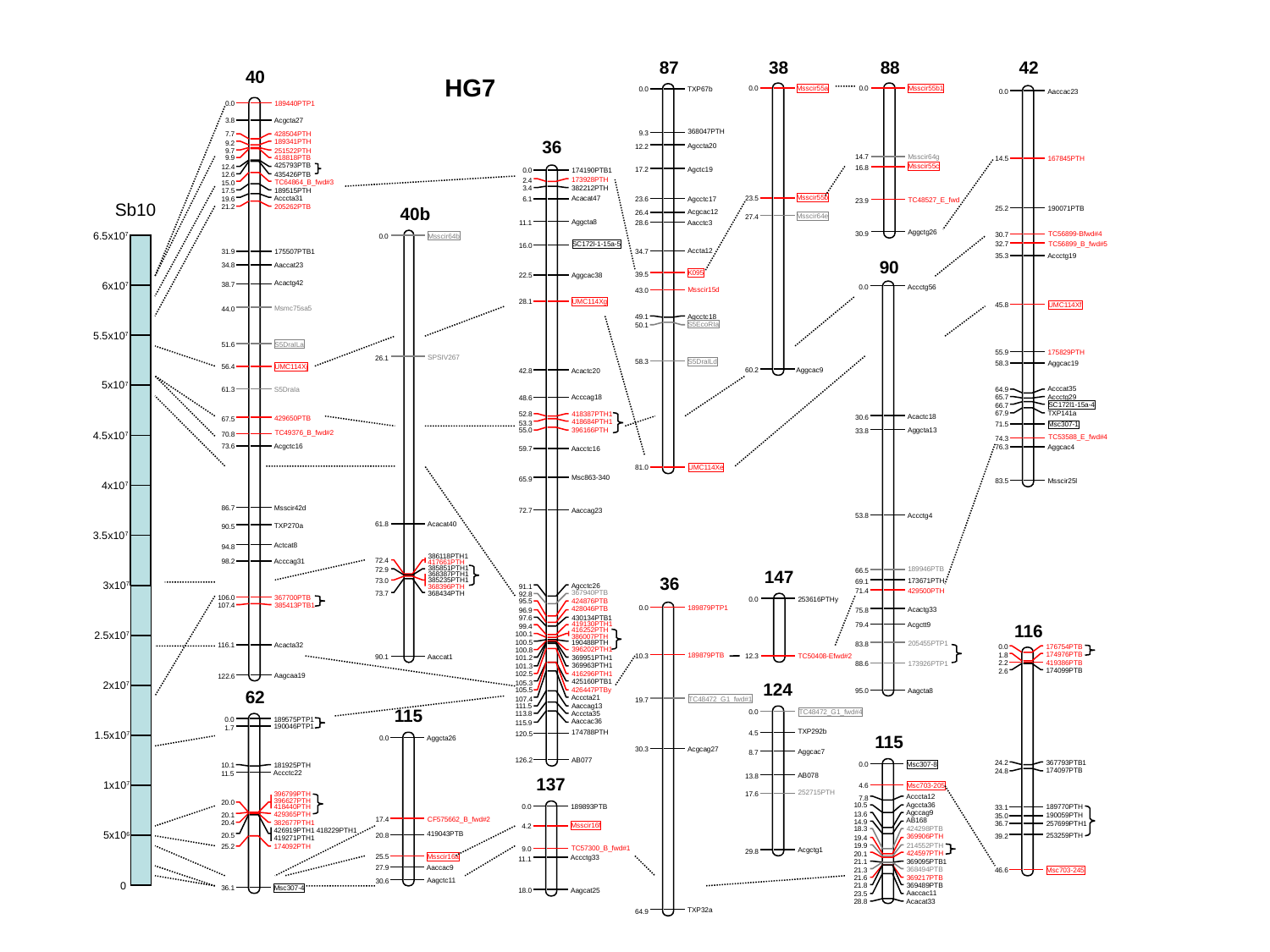

87
0.0
TXP67b
368047PTH
9.3
Agccta20
12.2
17.2
Agctc19
23.6
Agcctc17
Acgcac12
26.4
28.6
Aacctc3
Accta12
34.7
K095
39.5
Msscir15d
43.0
49.1
Agcctc18
S5EcoRIa
50.1
58.3
S5DraILd
81.0
UMC114Xe
38
0.0
Msscir55a
Msscir55b
23.5
Msscir64e
27.4
60.2
Aggcac9
88
0.0
Msscir55b1
14.7
Msscir64g
Msscir55c
16.8
TC48527_E_fwd
23.9
Aggctg26
30.9
42
0.0
Aaccac23
14.5
167845PTH
25.2
190071PTB
TC56899-Bfwd#4
30.7
32.7
TC56899_B_fwd#5
35.3
Accctg19
45.8
UMC114Xf
55.9
175829PTH
58.3
Aggcac19
Acccat35
64.9
65.7
Accctg29
SC172l1-15a-4
66.7
67.9
TXP141a
71.5
Msc307-1
TC53588_E_fwd#4
74.3
76.3
Aggcac4
83.5
Msscir25l
HG7
40
0.0
189440PTP1
3.8
Acgcta27
7.7
428504PTH
189341PTH
9.2
9.7
251522PTH
9.9
418818PTB
425793PTB
12.4
12.6
435426PTB
TC64864_B_fwd#3
15.0
17.5
189515PTH
Acccta31
19.6
21.2
205262PTB
31.9
175507PTB1
34.8
Aaccat23
Acactg42
38.7
Msmc75sa5
44.0
51.6
S5DraILa
56.4
UMC114Xj
61.3
S5DraIa
429650PTB
67.5
TC49376_B_fwd#2
70.8
73.6
Acgctc16
86.7
Msscir42d
TXP270a
90.5
Actcat8
94.8
98.2
Acccag31
106.0
367700PTB
107.4
385413PTB1
116.1
Acacta32
Aagcaa19
122.6
36
0.0
174190PTB1
173928PTH
2.4
3.4
382212PTH
Sb10
Acacat47
6.1
40b
Aggcta8
11.1
6.5x10^7^
0.0
Msscir64b
SC172l-1-15a-5
16.0
90
0.0
Accctg56
Acactc18
30.6
Aggcta13
33.8
53.8
Accctg4
189946PTB
66.5
173671PTH
69.1
71.4
429500PTH
Acactg33
75.8
79.4
Acgctt9
205455PTP1
83.8
88.6
173926PTP1
95.0
Aagcta8
22.5
Aggcac38
6x10^7^
28.1
UMC114Xg
5.5x10^7^
SPSIV267
26.1
42.8
Acactc20
5x10^7^
Acccag18
48.6
52.8
418387PTH1
418684PTH1
53.3
4.5x10^7^
55.0
396166PTH
59.7
Aacctc16
4x10^7^
Msc863-340
65.9
72.7
Aaccag23
61.8
Acacat40
3.5x10^7^
386118PTH1
72.4
417661PTH
385851PTH1
72.9
147
0.0
253616PTHy
12.3
TC50408-Efwd#2
368387PTH1
3x10^7^
36
0.0
189879PTP1
189879PTB
10.3
TC48472_G1_fwd#1
19.7
30.3
Acgcag27
TXP32a
64.9
385235PTH1
73.0
Agcctc26
368396PTH
91.1
367940PTB
73.7
368434PTH
92.8
95.5
424876PTB
428046PTB
96.9
97.6
430134PTB1
116
0.0
176754PTB
174976PTB
1.8
2.2
419386PTB
174099PTB
2.6
24.2
367793PTB1
174097PTB
24.8
189770PTH
33.1
190059PTH
35.0
36.7
257699PTH1
253259PTH
39.2
46.6
Msc703-245
419130PTH1
2.5x10^7^
99.4
416252PTH
100.1
386007PTH
100.5
190488PTH
396202PTH1
100.8
90.1
Aaccat1
101.2
369951PTH1
369963PTH1
101.3
102.5
416296PTH1
2x10^7^
425160PTB1
105.3
124
0.0
TC48472_G1_fwd#4
TXP292b
4.5
Aggcac7
8.7
AB078
13.8
252715PTH
17.6
Acgctg1
29.8
62
105.5
426447PTBy
Acccta21
107.4
111.5
Aaccag13
115
0.0
Aggcta26
17.4
CF575662_B_fwd#2
419043PTB
20.8
25.5
Msscir16a
27.9
Aaccac9
Aagctc11
30.6
113.8
Acccta35
0.0
189575PTP1
Aaccac36
115.9
1.5x10^7^
190046PTP1
1.7
174788PTH
120.5
115
0.0
Msc307-8
4.6
Msc703-205
Acccta12
7.8
10.5
Agccta36
Agccag9
13.6
AB168
14.9
18.3
424298PTB
369906PTH
19.4
19.9
214552PTH
424597PTH
20.1
21.1
369095PTB1
368494PTB
21.3
21.6
369217PTB
21.8
369489PTB
Aaccac11
23.5
28.8
Acacat33
126.2
AB077
10.1
181925PTH
Accctc22
11.5
1x10^7^
137
0.0
189893PTB
Msscir16f
4.2
TC57300_B_fwd#1
9.0
Accctg33
11.1
18.0
Aagcat25
396799PTH
396627PTH
20.0
418440PTH
429365PTH
20.1
20.4
382677PTH1
5x10^6^
426919PTH1
418229PTH1
20.5
419271PTH1
25.2
174092PTH
0
36.1
Msc307-4

## Slide 8
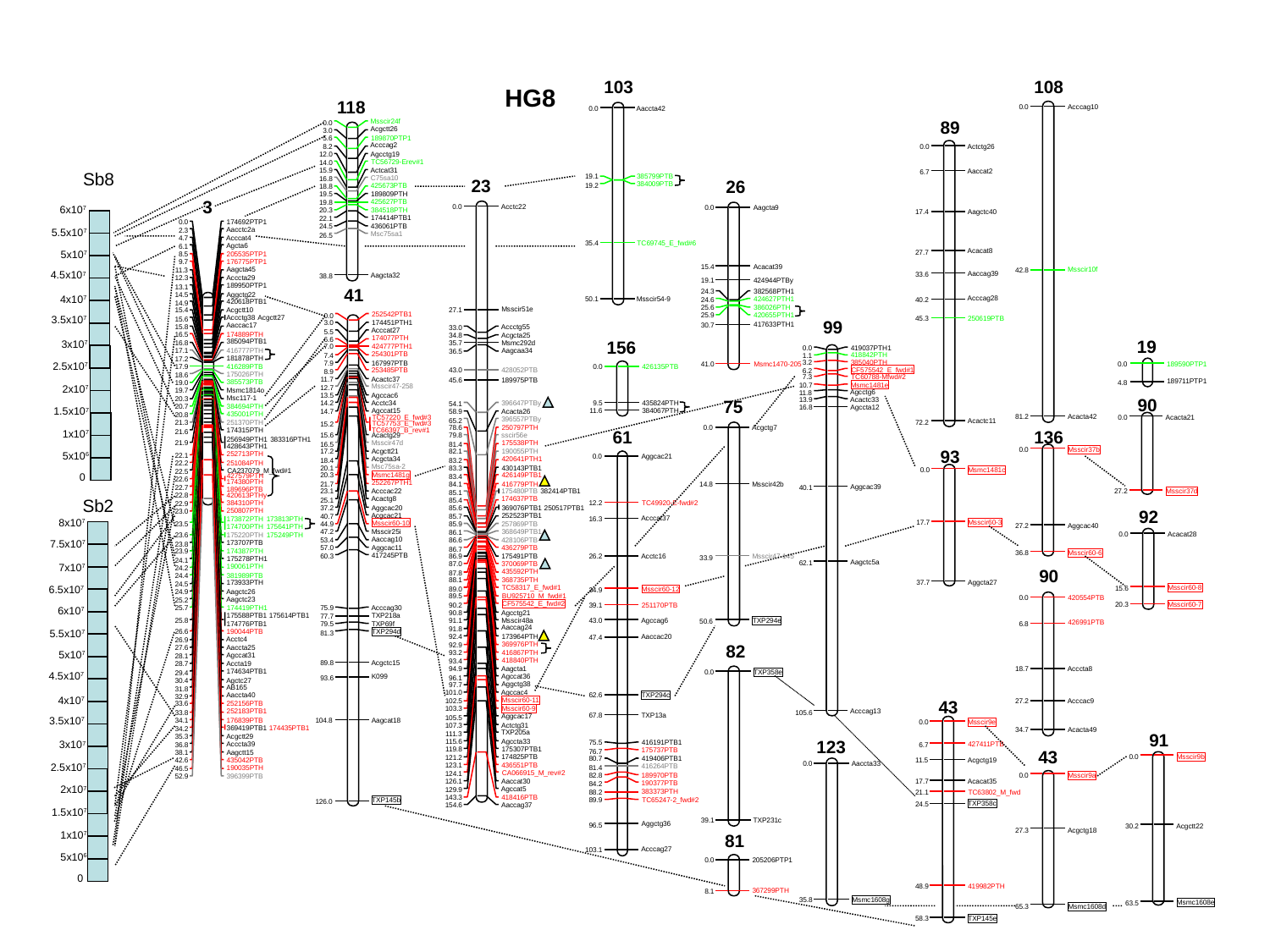

HG8
103
0.0
Aaccta42
19.1
385799PTB
384009PTB
19.2
35.4
TC69745_E_fwd#6
50.1
Msscir54-9
108
0.0
Acccag10
Msscir10f
42.8
81.2
Acacta42
118
Msscir24f
0.0
Acgctt26
3.0
5.6
189870PTP1
Acccag2
8.2
12.0
Agcctg19
TC56729-Erev#1
14.0
15.9
Actcat31
C75sa10
16.8
425673PTB
18.8
19.5
189809PTH
425627PTB
19.8
20.3
384518PTH
174414PTB1
22.1
24.5
436061PTB
Msc75sa1
26.5
Aagcta32
38.8
89
0.0
Actctg26
Aaccat2
6.7
17.4
Aagctc40
Acacat8
27.7
Aaccag39
33.6
Acccag28
40.2
45.3
250619PTB
Acactc11
72.2
Sb8
23
0.0
Acctc22
Msscir51e
27.1
Accctg55
33.0
34.8
Acgcta25
35.7
Msmc292d
Aagcaa34
36.5
43.0
428052PTB
45.6
189975PTB
396647PTBy
54.1
58.9
Acacta26
396557PTBy
65.2
78.6
250797PTH
79.8
sscir56e
175538PTH
81.4
82.1
190055PTH
420641PTH1
83.2
83.3
430143PTB1
426149PTB1
83.4
84.1
416779PTH
175480PTB
382414PTB1
85.1
174637PTB
85.4
85.6
369076PTB1
250517PTB1
252523PTB1
85.7
85.9
257869PTB
368649PTB1
86.1
86.6
428106PTB
436279PTB
86.7
86.9
175491PTB
87.0
370069PTB
435592PTH
87.8
88.1
368735PTH
TC58317_E_fwd#1
89.0
89.5
BU925710_M_fwd#1
CF575542_E_fwd#2
90.2
90.8
Agcctg21
91.1
Msscir48a
Aaccag24
91.8
92.4
173964PTH
369976PTH
92.9
93.2
416867PTH
418840PTH
93.4
94.9
Aagcta1
Agccat36
96.1
Aggctg38
97.7
101.0
Agccac4
Msscir60-11
102.5
103.3
Msscir60-9
Aggcac17
105.5
107.3
Actctg31
TXP205a
111.3
115.6
Agccta33
119.8
175307PTB1
174825PTB
121.2
123.1
436551PTB
CA066915_M_rev#2
124.1
126.1
Aaccat30
Agccat5
129.9
143.3
418416PTB
154.6
Aaccag37
26
0.0
Aagcta9
15.4
Acacat39
19.1
424944PTBy
24.3
382568PTH1
424627PTH1
24.6
25.6
386026PTH
25.9
420655PTH1
417633PTH1
30.7
41.0
Msmc1470-205
3
6x10^7^
0.0
174692PTP1
5.5x10^7^
Aacctc2a
2.3
4.7
Acccat4
5x10^7^
Agcta6
6.1
8.5
205535PTP1
9.7
176775PTP1
4.5x10^7^
Aagcta45
11.3
12.3
Acccta29
189950PTP1
13.1
41
252542PTB1
0.0
3.0
174451PTH1
Acccat27
5.5
174077PTH
6.6
7.0
424777PTH1
254301PTB
7.4
7.9
167997PTB
253485PTB
8.9
11.7
Acactc37
Msscir47-258
12.7
13.5
Agccac6
14.2
Acctc34
Agccat15
14.7
TC57220_E_fwd#3
TC57753_E_fwd#3
15.2
TC66397_B_rev#1
15.6
Acactg29
Msscir47d
16.5
17.2
Acgctt21
Acgcta34
18.4
Msc75sa-2
20.1
20.3
Msmc1481g
252267PTH1
21.7
23.1
Acccac22
Acactg8
25.1
37.2
Aggcac20
Acgcac21
40.7
Msscir60-10
44.9
47.2
Msscir25i
Aaccag10
53.4
57.0
Aggcac11
417245PTB
60.3
75.9
Acccag30
TXP218a
77.7
79.5
TXP69f
TXP294d
81.3
89.8
Acgctc15
K099
93.6
104.8
Aagcat18
TXP145b
126.0
4x10^7^
14.5
Aggctg22
420618PTB1
14.9
15.4
Acgctt10
3.5x10^7^
Accctg38
Acgctt27
15.6
99
0.0
419037PTH1
418842PTH
1.1
3.2
385040PTH
CF575542_E_fwd#1
6.2
7.3
TC60788-Mfwd#2
10.7
Msmc1481e
Agcctg6
11.8
13.9
Acactc33
16.8
Agccta12
Aggcac39
40.1
Aagctc5a
62.1
Acccag13
105.6
Aaccac17
15.8
16.5
174889PTH
3x10^7^
19
0.0
189590PTP1
189711PTP1
4.8
156
0.0
426135PTB
9.5
435824PTH
11.6
384067PTH
385094PTB1
16.8
17.1
416777PTH
2.5x10^7^
181878PTH
17.2
17.9
416289PTB
175026PTH
18.6
2x10^7^
385573PTB
19.0
19.7
Msmc1814o
Msc117-1
90
0.0
Acacta21
27.2
Msscir37d
20.3
75
0.0
Acgctg7
14.8
Msscir42b
Msscir47-145
33.9
TXP294e
50.6
1.5x10^7^
20.7
384694PTH
435001PTH
20.8
21.3
251370PTH
1x10^7^
174315PTH
136
0.0
Msscir37b
27.2
Aggcac40
36.8
Msscir60-6
61
0.0
Aggcac21
12.2
TC49920-E-fwd#2
Acccat37
16.3
26.2
Acctc16
Msscir60-12
34.9
39.1
251170PTB
43.0
Agccag6
Aaccac20
47.4
62.6
TXP294c
67.8
TXP13a
75.5
416191PTB1
175737PTB
76.7
80.7
419406PTB1
416264PTB
81.4
82.8
189970PTB
190377PTB
84.2
383373PTH
88.2
89.9
TC65247-2_fwd#2
Aggctg36
96.5
Acccag27
103.1
21.6
256949PTH1
383316PTH1
21.9
428643PTH1
5x10^6^
93
0.0
Msmc1481c
17.7
Msscir60-3
37.7
Aggcta27
252713PTH
22.1
22.2
251084PTH
0
CA237079_M_fwd#1
22.5
427579PTH
22.6
174380PTH
22.7
189696PTB
Sb2
22.8
420613PTHy
384310PTH
22.9
250807PTH
92
0.0
Acacat28
Msscir60-8
15.6
20.3
Msscir60-7
23.0
8x10^7^
173872PTH
173813PTH
23.5
174700PTH
175641PTH
7.5x10^7^
23.6
175220PTH
175249PTH
173707PTB
23.8
23.9
174387PTH
7x10^7^
175278PTH1
24.1
190061PTH
24.2
90
0.0
420554PTB
426991PTB
6.8
18.7
Acccta8
27.2
Acccac9
34.7
Acacta49
24.4
381989PTB
6.5x10^7^
173933PTH
24.5
24.9
Aagctc26
Aagctc23
25.2
6x10^7^
25.7
174419PTH1
175588PTB1
175614PTB1
25.8
174776PTB1
5.5x10^7^
26.6
190044PTB
Acctc4
26.9
82
0.0
TXP358e
39.1
TXP231c
5x10^7^
27.6
Aaccta25
Agccat31
28.1
28.7
Accta19
4.5x10^7^
174634PTB1
29.4
30.4
Agctc27
AB165
31.8
4x10^7^
Aaccta40
32.9
43
0.0
Msscir9e
427411PTB
6.7
11.5
Acgctg19
17.7
Acacat35
21.1
TC63802_M_fwd
TXP358c
24.5
48.9
419982PTH
58.3
TXP145e
33.6
252156PTB
252183PTB1
3.5x10^7^
33.8
34.1
176839PTB
369419PTB1
174435PTB1
34.2
91
0.0
Msscir9b
30.2
Acgctt22
Msmc1608e
63.5
3x10^7^
35.3
Acgctt29
123
0.0
Aaccta33
35.8
Msmc1608g
Acccta39
36.8
43
0.0
Msscir9a
27.3
Acgctg18
65.3
Msmc1608d
38.1
Aagctt15
2.5x10^7^
42.6
435042PTB
190035PTH
46.5
52.9
396399PTB
2x10^7^
1.5x10^7^
1x10^7^
81
0.0
205206PTP1
367299PTH
8.1
5x10^6^
0
